# Impact of valproic acid on busulfan pharmacokinetics: *In vitro* assessment of potential drug-drug interaction

**DOI:** 10.1371/journal.pone.0280574

**Published:** 2023-01-25

**Authors:** Bashayer F. Al-Enezi, Nada Al-Hasawi, Kamal M. Matar

**Affiliations:** 1 Department of Pharmacology & Therapeutics, Faculty of Pharmacy, Kuwait University, Kuwait City, Kuwait; 2 Department of Pharmaceutical Chemistry, Faculty of Pharmacy, Kuwait University, Kuwait City, Kuwait; GLA University, INDIA

## Abstract

Busulfan (Bu) is an alkylating agent commonly used at high doses in the preparative regimens of hematopoietic stem cell transplantation (HSCT). It has been shown that such high doses of Bu are associated with generalized seizures which are usually managed by prophylactic antiepileptic drugs (AEDs) such as valproic acid (VPA). Being a strong enzyme inhibitor, VPA may inhibit Bu metabolism and thus increase its potential toxicity. Despite its clinical relevance, the potential interaction between Bu and VPA has not yet been evaluated. The aim of the present study was to assess and evaluate the potential drug-drug interaction (DDI) between Bu and VPA. This study was carried out by incubating Bu in laboratory-prepared rat liver-subcellular fractions including S9, microsomes, and cytosol, alone or in combination with VPA. The liver fractions were prepared by differential centrifugation of the liver homogenate. Analysis of Bu was employed using a fully validated LC-MS/MS method. The validation parameters were within the proposed limits of the international standards guidelines. Bu metabolic stability was assessed by incubating Bu at a concentration of 8 μg/ml in liver fractions at 37°C. There were significant reductions in Bu levels in S9 and cytosolic fractions, whereas these levels were not significantly (P ˃ 0.05) changed in microsomes. However, in presence of VPA, Bu levels in S9 fraction remained unchanged. These results indicated, for the first time, the potential metabolic interaction of Bu and VPA being in S9 only. This could be explained by inhibiting Bu cytosolic metabolism by the interaction with VPA either by sharing the same metabolic enzyme or the required co-factor. In conclusion, the present findings suggest, for the first time, a potential DDI between Bu and VPA *in vitro* using rat liver fractions. Further investigations are warranted in human-derived liver fractions to confirm such an interaction.

## 1. Introduction

Busulfan (Bu) is a bi-functional alkylating agent, chemically named 1,4-butanediol-dimethanesulfonate. In its action, Bu interferes with DNA replication and transcription processes, which prevent cellular proliferation and differentiation [1]. In the early 1950s, Bu was initially identified as a palliative treatment of chronic myeloid leukemia (CML) [2], however, its therapeutic use was gradually shifted to pre-transplant conditioning due to its potent myeloablative effect [3]. Since the 1980s, Bu was considered to be one of the most commonly used drugs in hematopoietic stem cell transplantation (HSCT) conditioning regimens at a total dose of 3.2–16 mg/kg over 1–4 days to reduce graft rejection [4, 5]. Moreover, the IV formulation was approved by FDA in 1999 to minimize the extreme pharmacokinetic variabilities experienced with an oral Bu [1, 6].

**Fig 1:**
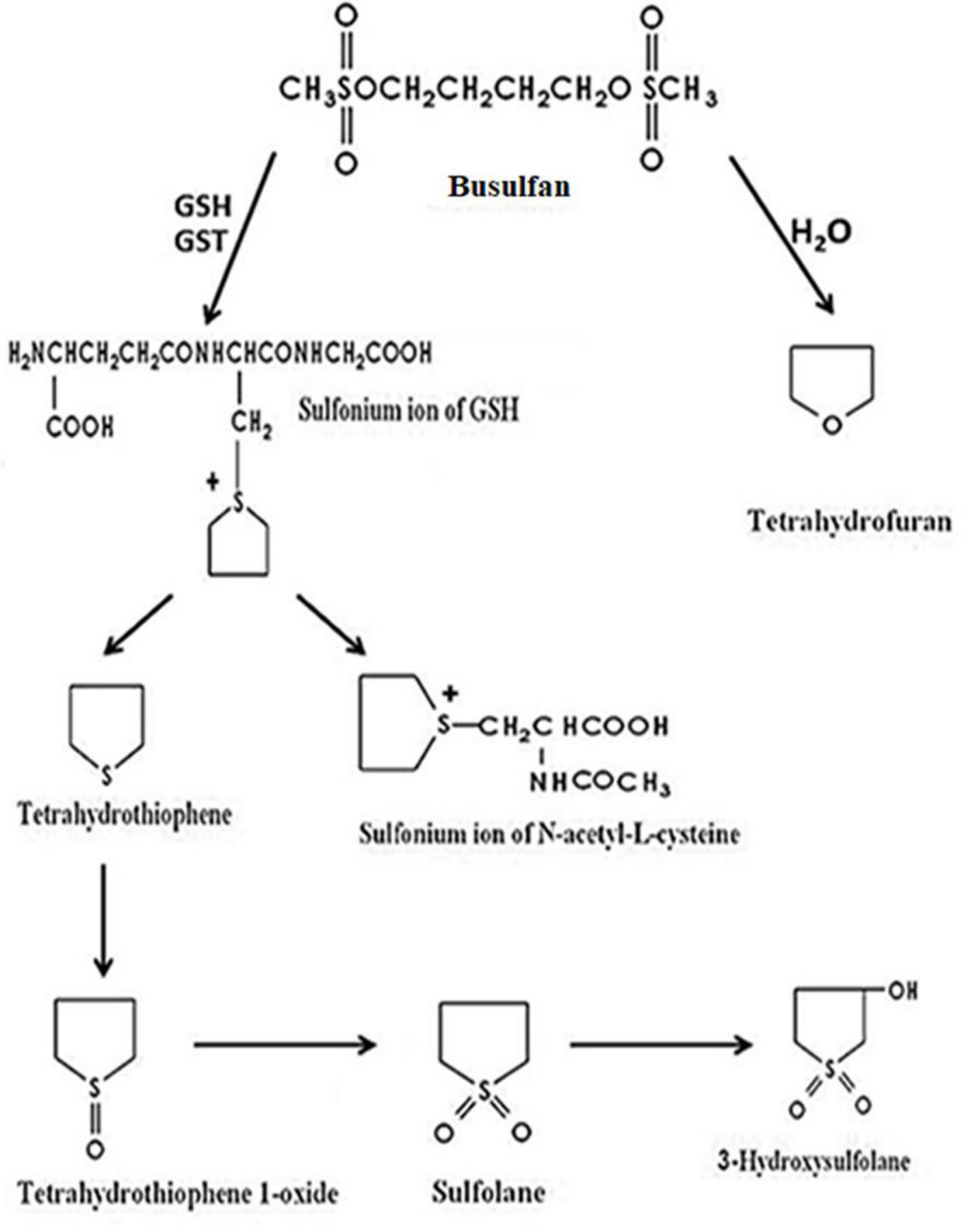
Busulfan metabolic pathway (El-Serafi et al., 2017).

Despite being in clinical use for a long time, the complete metabolic profile of Bu has not yet been determined. The common Bu metabolic pathway that has been recently identified as illustrated in Fig 1, is extensively metabolized in the liver with approximately 2% being excreted unchanged in urine [7]. The major metabolic process of Bu is conjugation with glutathione (GSH) by glutathione-s-transferase (GST) enzymes, mainly GSTA1, where this pathway accounts for 50% of Bu elimination from the body [8]. Another minor pathway involves Bu hydrolysis either non-enzymatically or possibly mediated by hydrolase enzymes [9]. A well-identified Bu metabolite, methane sulfonic acid, is formed as a by-product of the two pathways was detected in blood and many other tissues in rats [10]. In Bu conjugation with GSH, a positively charged sulfonium ion is formed and further metabolized either through mercapturic acid pathway producing Bu N-acetylated cysteine conjugate, or the sulfonium which is subjected to β-elimination reaction with a formation of tetrahydrothiophene (THT). The resulting THT undergoes serial metabolic oxidation reactions, hypothetically catalyzed by cytochrome-P450 enzymes (CYPs) or flavin-containing monooxygenase (FMO), which produces tetrahydrothiophene 1-oxide (THT 1-oxide) which is further oxidized to sulfolane and 3-hydroxysulfolane [9, 11, 12]. Lately, these metabolites were also detected in plasma and urine of patients given high doses of Bu [7, 13]. This indicates that Bu metabolic process is similar in both humans and rats.

The association between Bu conditioning regimens and relevant complications is mainly related to the narrow therapeutic window of the drug. Thus, the area under the plasma Bu concentration-time curve (AUC) ranging from 900 to 1500 μM.min is considered adequate for optimal dosing of Bu [14, 15]. Consequently, very low Bu dose has been correlated with graft failure, disease recurrence and shortened survival rate. In contrast, high Bu systemic exposure is associated with increased toxicities in which neurotoxicity is the most serious one [1, 16–18]. Neurotoxicity manifested primarily as generalized seizures, which results from administering Bu at a dose ˃ 600/m^2^ or 16 mg/kg. Seizures usually experienced between the second day of Bu administration and within the 24 h following the final Bu dose [5]. It has been attributed to the ability of Bu to cross the blood-brain barrier, which is enhanced greatly by low protein binding property, and thus demonstrating comparable levels in plasma and cerebrospinal fluid [14].

To control seizures, various antiepileptic drugs (AEDs) have been utilized as prophylaxis during Bu conditioning. The ideal prophylactic AED should be safe and doesn’t interact with the conditioning regimen or enhance its toxicity. Phenytoin has historically been the AED of choice that effectively reduces the prevalence of Bu-induced seizures to about 0–5.5%. However, due to phenytoin’s action as a potent enzyme inducer, drug-drug interaction (DDI) raised, which leads to enhanced Bu clearance and consequently decreased its plasma levels. Thus, phenytoin has been replaced by other AEDs [19–21]. On the other hand, levetiracetam, a second-generation AED, neither induces nor inhibits CYP450 metabolizing enzymes rendering it an ideal alternative to phenytoin in preventing Bu-induced seizures [5, 21, 22].

Valproic acid (VPA) is a conventional AED which has also used for prophylaxis of Bu-induced seizures at a dose of 30 mg/kg/day starting 24 h prior to the first Bu dose and continuing until 24 h after the last Bu dose [23, 24]. Using VPA for this indication is not a common practice worldwide. However, this procedure is widely practiced in Kuwait despite the fact that safely administration of VPA before Bu therapy has not yet been confirmed. Therefore, carrying out DDI study to investigate the potential interaction between these two drugs is of high clinical relevance. Interestingly, it has been reported that VPA failed to prevent Bu-induced seizures in mice model. Therefore, due to some doubts about VPA effectiveness as a prophylactic AED and its documented toxicities such as hematologic and hepatic toxicities, it is not recommended to be used for this indication [5].

The main rationale for conducting this study is based on the fact that VPA is a broad-spectrum enzyme inhibitor that inhibits the activity of many drug metabolizing enzymes. It mainly inhibits the activity of CYP2C9 and also exhibits minimal inhibitory effects on CYP2C19 and CYP3A4 [25–27]. As a result, metabolism of the concomitantly prescribed drugs would be reduced with a potential of higher serum concentrations leading to a likelihood of toxicity. Although Bu is mainly metabolised by GST enzyme, many studies have reported serious DDIs between Bu and CYP inhibitors such as itraconazole and metronidazole, and CYP inducers including phenytoin [28–31]. This might be explained by affecting the oxidation process of Bu metabolites; THT, THT 1-oxide and sulfolane, where CYPs enzymes were shown to have appreciable contribution. Consequently, the metabolism of Bu might also be influenced by such agents including VPA, a well-known CYPs inhibitor. Apart from being a strong inhibitor of several CYP enzymes, VPA is known to be a substrate for GST, the major Bu metabolizing enzyme [32, 33], which increases the potential of having DDI. Therefore, it is expected that VPA may affect Bu pharmacokinetics by inhibiting its metabolism resulting in lower Bu metabolic clearance with a potential toxicity. For these reasons, along with the fact that Bu metabolic pathway has not been well-defined, the demand for conducting such study to evaluate the effect of VPA on Bu metabolism and assess the *in vitro* potential pharmacokinetic DDI between the two medications. The objective of the present study was to set up *in vitro* models for assessing the metabolism of Bu alone and in combination with VPA and hence for investigating the metabolic impact of VPA on Bu in rat liver fractions.

## 2. Materials and methods

### 2.1. Chemicals and reagents

Unless otherwise specified, all reagents and chemicals were purchased from Sigma Aldrich Chemical Co. (St. Louis MO, USA). Busulfan-d8 (Bu-D8) internal standard was supplied by Santa Cruz Biotechnology, Inc. (Dallas TX, California).

### 2.2. Instrumentation and LC-MS/MS conditions for Bu determination

The chromatographic separation of the analyte was performed on Symmetry^®^ C_18_ column (5 μm, 3.9 mm x 50 mm) and protected by a pre-column filter of the same packing material. Bu was analyzed employing a modification of a previously described LC-MS/MS method [34]. The Liquid chromatographic system consisted of Waters Alliance e2695, solvent delivery system and an autosampler (Waters Assoc., Milford, MA, USA). Ionization source was achieved through electrospray ionization (ESI) of triple quadrupole tandem mass spectrometer (Quattro-micro LC-MS/MS, Waters Assoc., Milford, USA). The mobile phase consisted of 20 mM NH_4_Ac/MeOH (20:80, v/v) was used and run at a flow rate of 0.2 ml/min under isocratic conditions. Data acquisition was performed by MassLynx Software (version 4.1, Micromass, Manchester, UK).

#### 2.2.1. Standard solutions, calibration standards and quality control (QC) samples

Stock solutions of Bu (MW: 246.3 g/mol) and the IS (MW: 254.4 g/mol) were prepared in ACN at a final concentration of 1 mg/ml. The prepared stock solutions were aliquoted and stored at -80°C until use. The stock solutions were further diluted with ACN to yield 100 μg/ml Bu and 10 μg/ml IS working standard solutions.

The calibration standards of Bu were prepared by spiking a 1.0 ml of drug-free rat plasma with Bu working solution at concentrations of 1, 2, 3, 6, 7.5 and 10 μg/ml. Similarly, QC samples were prepared in drug-free rat plasma at concentrations of 1.5, 5 and 8 μg/ml, which cover the lower, middle and higher limits of the calibration. The spiked plasma samples were then aliquoted (100 μl) and kept frozen at -80°C pending analysis.

#### 2.2.2. Sample preparation

The frozen spiked rat plasma samples, including calibrators or QC samples, were thawed at ambient temperature. From each sample, a 100 μl aliquot was transferred to a 2.0 ml Eppendorf tube then 20 μl of the IS (10 μg/ml) was added to each tube and vortex-mixed for 30 sec. After that, 150 μl of saturated NaCl (26%) solution was added and the tubes were vortex-mixed for 30 sec. Liquid-liquid extraction (LLE) was achieved by adding 1.5 ml *ter*-butyl ethyl ether followed by 30 sec vortex-mixing and 15 min shaking by rotary mixer; adjusted at 50 rpm. The tubes were then centrifuged at 10,000 x *g* for 10 min and the resultant organic layers were separated and evaporated under purified nitrogen gas stream. The resultant residues were reconstituted into 150 μl mobile phase, vortex-mixed and centrifuged at 10,000 x *g* for 10 min. Finally, a 100 μl of the supernatant was transferred to the inserts of autosampler vials and 10 μl was injected into the LC-MS system for analysis.

#### 2.2.3. Method validation

After optimizing LC-MS/MS conditions, the method was validated according to international standards as recommended by Food and Drug Administration [35] and European Medicines Agency [36]. The assessed validation parameters included linearity, selectivity, accuracy, precision, matrix effect, stability and carryover.

*2*.*2*.*3*.*1*. *Linearity and lower limit of quantification (LLOQ)*. Linearity of the calibration curve demonstrates the relationship between instrument response and known concentrations of the analyte using IS method. Standard curves were constructed by assaying drug-free plasma samples in replicates of six, a zero sample and the six prepared calibrators in the actual range of 1–10 μg/ml. Calibrator concentrations were then plotted against the response to get the calibration curve with slope, intercept and correlation coefficient (*r*) using the least squares linear regression model. Various parameters of the obtained regression equation were automatically calculated byMassLynx software.

*2*.*2*.*3*.*2*. *Selectivity*. The ability of the analytical method to distinguish and quantify the analyte in the presence of other components in the sample was investigated. This was achieved by analyzing drug-free plasma samples from ten different rats for potential interferences. The mass detector response at the retention times of Bu and IS was assessed and compared to that of spiked plasma samples at LLOQ, 1 μg/ml.

*2*.*2*.*3*.*3*. *Accuracy and precision*. Accuracy of the method describes the closeness of mean test results obtained by the method to the true concentration of the analyte, whereas the precision describes the closeness of individual measures of an analyte when the procedure is carried out repeatedly to multiple aliquots of the same biological matrix sample. Accuracy and precision were estimated through the intra-run and inter-run variability analysis, by analyzing the three levels of quality control samples (QC1, QC2 and QC3) in addition to the LLOQ in sets of replicates. In case of intra-day accuracy and precision, the QC samples and the LLOQ were assessed in quintuplicate analyses from one calibration curve batch in one day. However, in case of inter-run precision and accuracy, the QC samples and the LLOQ were assessed from five independent runs over a period of one month.

Accuracy was expressed as the percent deviation from the nominal concentration (Bias%), whereas the relative standard deviation (RSD%) served as a measure of the method’s precision. The developed method is considered to be accurate and precise when the calculated Bias% and RSD% are ≤15% for the quality control samples, except for the LLOQ which should be ≤20%.

*2*.*2*.*3*.*4*. *Matrix effect*. Matrix effect is defined as the potential change in the sensitivity of the bioanalytical technique caused by co-eluting matrix components that might affect the ionization of the target analyte as well as its chromatographic response [37]. This could cause an ion suppression or enhancement which would ultimately affect the accuracy, reproducibility, and sensitivity of the quantification of analyte of interest [38]. Matrix effect was assessed by employing post-column infusion protocol [39]. Solutions of Bu and IS (20 μg/ml) were continuously infused into the eluent from the column through post column “tee” connector using Hamilton syringe pump at a flow rate of 20 μl/min. After which, an aliquot of 10 μl of drug-free rat plasma extract was analysed by chromatographic system. The ion intensities at *m/z* 264.4˃151.1 for Bu and *m/z* 272.3˃159 for IS were used to assess the ion suppression or enhancement.

*2*.*2*.*3*.*5*. *Stability*. Several types of stability tests were carried out in this work. These include freeze-thaw stability, short-term stability and long-term stability. Freeze-thaw stability was assessed by storing aliquots of each concentration level of QC samples (QC1, QC2 and QC3) at the intended storage temperature (-80°C) for 24 h and then thawing them at RT. Once completely thawed, aliquots were analyzed by the LC-MS/MS method while the other samples were frozen again under the same conditions. The freeze–thaw cycle was repeated five times, and in each time, frozen samples were allowed to stand at RT for 2 h before being prepared for analysis. Short-term stability was assessed by analyzing five aliquots of each QC levels at RT for up to 2 h, which is the time exceeding their residential duration in autosampler. In long-term stability test, the test was achieved by storing aliquots of the three QC levels under the same conditions as the study samples (-80°C) and analyzing them at different occasions up to one month. The concentrations of all the stability samples were compared to the nominal QC levels.

*2*.*2*.*3*.*6*. *Carryover*. Carryover was assessed by injecting blank sample followed by injecting the upper limit of quantification (ULOQ) sample (10 μg/ml), then re-injecting the same blank sample. This was repeated with five different blank samples and the obtained chromatograms of the blank and ULOQ samples were evaluated for any carryover effect.

### 2.3. Ethics statement

The present study was approved by Ethics Committee for “Animal Care and Use” of Health Science Center, Kuwait University. All experimental protocols were conducted according to the international standards of animal care of “Helsinki Declaration for Ethical Principles of Medical Research”.

### 2.4. Preparation of biological fluids and protein determination

The metabolic stability of Bu in biological fluids was determined by incubating the drug in rat plasma and liver fractions (S9, microsomes and cytosolic fractions). The biological fluids were prepared from Eight Male Sprague Dawley (MSD) rats (approximately 3 months of age) with body weights raged from 350 to 450 g. The rats were anesthetized by exposing them to halothane for less than 2 min. Blood samples were then collected from the heart using 1 ml syringes through a longitudinal opening over the chest while the heart was still beating to promote blood flow [40]. The collected blood samples were transferred into Eppendorf tubes containing 10 μl of heparinized saline solution. The blood samples were then centrifuged for 10 minutes at 10,000 x *g* and the resulting plasma samples were separated, aliquoted and stored at -80°C.

To prepare liver fractions, liver samples of the eight rats were processed individually in a separate day to permit for rapid handling while being kept on ice to maintain enzymes’ activities. Livers of the sacrificed rats were immediately excised, freed from fat and connective tissues, and perfused properly with PBS (pH 7.4). The livers were then divided into small pieces where each piece was weighed and placed into a plastic tube. Liver pieces were then homogenized in four volumes of their weights in PBS solution [41] using tissue homogenizer. The obtained liver homogenates were centrifuged at 9000 x *g* for 20 min at 4°C and the resulting supernatants (S9 fractions) were aliquoted (3 ml per aliquot), and the remaining S9 was further centrifuged for an hour at 100,000 _X_ *g* at 4°C. The resultant pellets were the microsomes (contain phase I enzymes), whereas supernatants were cytosols (contain phase II enzymes). The cytosols were immediately aliquoted (3 ml per aliquot), whereas the microsomes were resuspended in PBS and similarly aliquoted (0.5 ml per aliquot). The aliquoted liver fractions were kept at -80°C and used within 2 months from their preparation.

Protein concentrations of the prepared liver fractions were determined by Bradford assay [42, 43]. Briefly, serial dilutions of BSA were prepared in the range of 0–1.0 mg/ml in deionized water. Samples of liver fractions (prepared in PBS, pH 7.4) were diluted in deionized water at a ratio of 1:50 or 1:60. Blanks were similarly prepared by diluting PBS in deionized water at a ratio of 1:50 or 1:60 PBS. A volume of 3 ml Bradford reagent was added to blanks, BSA standard solutions and liver samples. Absorbance of the samples was measured at λ_max_ 595 nm using a UV-visible spectrophotometer within 5 to 60 min after the addition of Bradford reagent. Readings were recorded in triplicates and a linear standard curve was established by plotting the mean absorbance values of BSA standard solutions against their corresponding concentrations. For the liver samples, protein concentrations were determined from the established standard curve, and then multiplying the yield values by the dilution factors.

### 2.5. Assessment of microsomal activity by EROD assay

**Fig 2:**
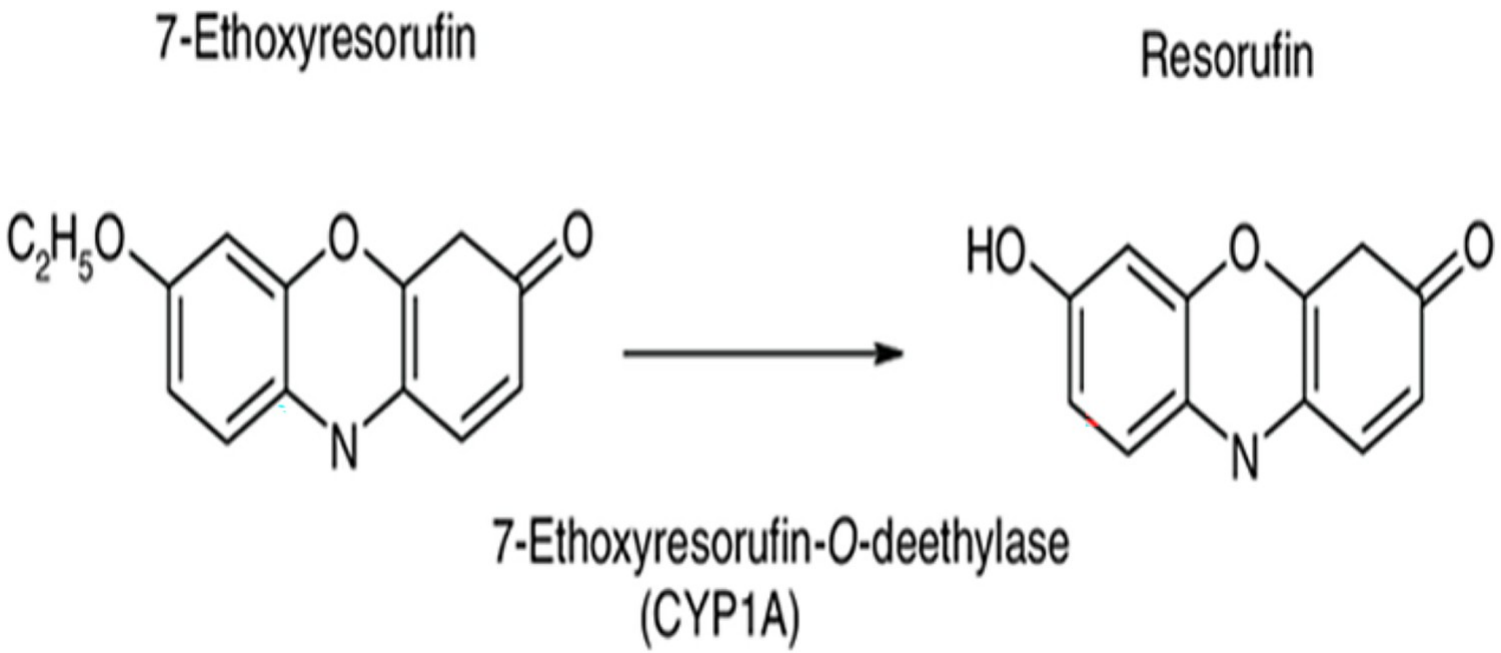
Oxidative-*o*-deethylation of 7-ER by active CYP1A1/2 to resorufin (Short and Springman, 2016).

The metabolic activity of the microsomes was assessed by the conversion of 7-Ethoxyresorufin (7-ER) to resorufin in the presence of active CYP1A1 enzymes [44]. This assessment was carried out by using a modified method of the previously described EROD assay [45]. The resulting resorufin was analyzed using high performance liquid chromatography with fluorescence detector (HPLC-FD), and the activity of CYP1A1 was expressed as ng resorufin formed/min/mg protein, Fig 2.

#### 2.5.1. Instrumentation and conditions of HPLC-FD for resorufin determination

Analysis of resorufin by HPLC was carried out using Waters Alliance system (e2695) connected to a multi-wavelength fluorescence detector (2475) (Waters Assoc., Milford, MS, USA). Chromatographic separation of resorufin was achieved by injecting 10 μl of the samples into a Symmetry^®^ C_18_ column (5 μm, 4.6 mm x 150 mm) kept at 40°C. Resorufin was eluted with a mobile phase consisting of PBS (pH 6.4)-MeOH-ACN (52:45:3 v/v), pH 7.3, which was delivered isocratically at a flow rate of 1.5 ml/min. The fluorescence detection was achieved at excitation and emission wavelengths of 560 and 585 nm, respectively. Resorufin was eluted at approximately 1 min, and data acquisition, processing and system control were achieved via Empower 2 software (Waters Assoc., Milford, MS, USA).

*2*.*5*.*1*.*1*. *Standard solutions*, *calibration standards and quality control samples*. Resorufin stock solution (100 μg/ml) was prepared by dissolving 2.6 mg resorufin in 26 ml of methanol. Aliquots (1 ml) of the stock solution were kept at -80°C and protected from light. Working solutions of resorufin (1 μg/ml) were freshly prepared by diluting the stock solution with PBS (pH 7.4)-methanol (50:50 v/v).

Resorufin calibration standards were prepared at concentrations of 10, 40, 60, 100, 120 and 160 ng/ml. The resorufin QC samples were prepared at concentrations of 20, 80 and 140 ng/ml to cover the lower, middle and higher range of the calibration curve. The calibration standards and QC samples were freshly prepared by spiking rat microsomes at protein concentration of 1.0 mg/ml with resorufin working solution at the proposed concentrations.

*2*.*5*.*1*.*2*. *Sample preparation*. Resorufin calibration standards and QC samples were prepared by protein precipitation (PPT) using ACN. The assay was initiated by preparing 1 ml of the solutions by spiking 1 ml of rat microsomes with resorufin working standard solution (1 μg/ml). After that, a 200 μl of each prepared standard was transferred to a clean 2 ml Eppendorf tube. After addition of 200 μl of ice-cold ACN, the samples were vortex-mixed for 30 sec. The samples were then rota-mixed at 50 rpm for 10 min and then centrifuged at 13,000 rpm for 10 min at 4°C. Finally, 100 μl of supernatants were transferred to the low volume inserts of autosampler and 10 μl was injected into the HPLC system. During the incubation process, the collected samples at each time point were then prepared by applying the same procedure that performed for calibration standards and QC samples and under the same experimental conditions.

*2*.*5*.*1*.*3*. *Method validation*. Once the fluorometric analytical method for resorufin determination has been developed, it was then validated for linearity, accuracy, precision, selectivity, stability and carryover. This again was carried out according to the standard guidelines [35, 36].

*2*.*5*.*1*.*3*.*1*. *Linearity and LLOQ*: Linearity of the fluorometric method was confirmed by applying external standard method. This was achieved by spiking the rat microsomes with resorufin (1 μg/ml) in the range of 10–160 ng/ml at six non-zero calibration standards. These standards were analyzed in replicates of six. Various parameters including slope, *y*-intercept, and correlation coefficient (*r*) were determined by the least squares linear regression model. Furthermore, the LLOQ (10 ng/ml) was also established by the same criteria indicated in Bu analytical method described above.

*2*.*5*.*1*.*3*.*2*. *Selectivity*: Selectivity of resorufin analytical method was assessed by analyzing six different lots of rat liver microsomes applying the same procedure described above for Bu analysis.

*2*.*5*.*1*.*3*.*3*. *Accuracy and precision*: Both intra-run and inter-run accuracy and precision were investigated using QC samples at three different concentrations, 20, 80 and 140 ng/ml. The intra-run accuracy and precision were assessed in one day by analyzing five replicates of QC samples in one calibration curve whereas, the inter-run accuracy and precision were determined by five replicates over a period of three weeks.

*2*.*5*.*1*.*3*.*4*. *Stability*: Samples collected in the actual EROD assay were analyzed immediately after being collected and processed during a period of not more than 2 h. Since the validation of resorufin stability in plasma and liver fractions is a function of the actual storage conditions that are applied in the study [35], short-term stability was investigated for resorufin in microsomes for a period exceeding the residential time of the samples in autosampler. This was achieved by analyzing five aliquots of each QC level at three different concentrations under the same conditions of the actual study. Analysis of samples was carried out at various time points up to 2 h. After that, the determined concentrations of each QC levels (analyzed at different time intervals) were compared to the nominal ones.

*2*.*5*.*1*.*3*.*5*. *Carryover*: Potential impact of previously injected sample on the analysis results of the following one was investigated employing the same procedure applied in Bu analytical method described above.

#### 2.5.2. EROD assay

A stock solution of 100 μg/ml 7-ER (MW: 241.24 g/mol) was prepared by dissolving 0.5 mg in 5 ml methanol. The stock solution was aliquoted into 2.0 ml Eppendorf polypropylene tubes that were protected from light and stored at -80°C. Solutions of exogenous cofactors which were required for the *in vitro* CYP1A1 activity, were freshly prepared. These were NADPH and MgCl_2_ at a concentration of 80 mM in 0.01 M PBS (pH 7.4).

Incubation of 7-ER in the microsomal fraction was carried out in duplicate. The prepared rat liver microsomes samples were de-frosted on ice and pooled together to yield pooled rat liver microsomes (PRLM). The incubations were carried out by adding 2 mg of PRLM into 2.0 ml Eppendorf tubes and completing the volume to 1890 μl with incubation buffer consisting of 1 mM EDTA, prepared in 0.01 M PBS (pH 7.4). The tubes were then placed in the shaking water bath (adjusted at 37°C) for 10 min. After that, 10 μl of 7-ER stock solution (100 μg/ml) was added to the pre-incubated microsomes samples, and the reaction was started by adding 50 μl of each cofactor solutions (NADPH and MgCl_2_). The final volume of the incubation mixture was 2 ml of 1 mg/ml of the microsomal protein, 0.5 μg/ml of 7-ER and 2 mM of each cofactor. A control sample was incubated and treated simultaneously, where it contained the same composition of the incubation mixture except for the cofactors which were replaced with PBS.

Immediately after adding the cofactors, a volume of 200 μl was withdrawn from the incubation mixture into a clean Eppendorf tube as 0-time sample. The samples were then withdrawn at time intervals of 5, 10, 20, 30, 45, 60, 90 and 120 min. A volume of 200 μl of cold ACN was then added to the withdrawn samples (to stop the enzymatic reaction) and vortex-mixed for 30 sec. After that, the samples were processed the same way as carried out with calibrators, and then analyzed by HPLC-FD and the concentration of the produced resorufin at each time point was determined based on the freshly prepared and assessed calibrators.

### 2.6. Assessment of cytosolic activity by GST assay

**Fig 3:**
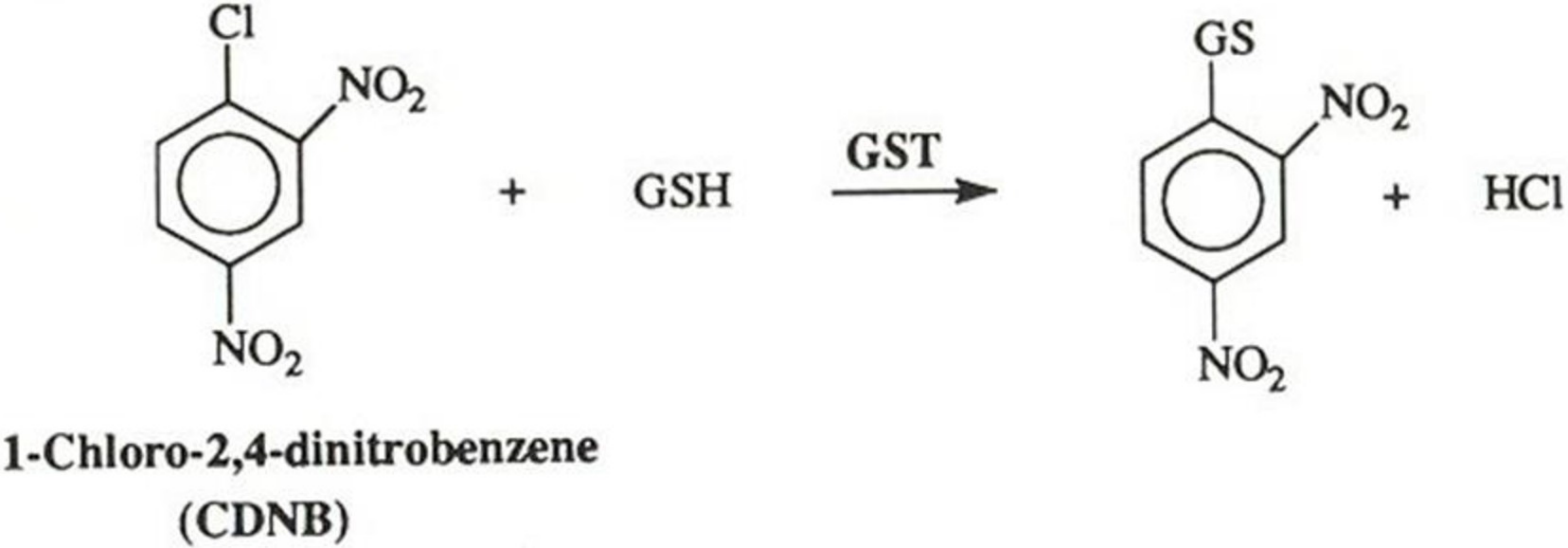
Conjugation of CDNB with GSH via active GST enzyme (Gonzalez, 2006).

The metabolic activity of cytosol was assessed by the conjugation reaction of 1-chloro-2,4-dinitrobenzene (CDNB) with glutathione (GSH) in presence of glutathione-S-transferase (GST) enzyme. This reaction produces a dinitrophenyl thioether that can be measured at λ_max_ 340 nm by using a spectrophotometer, Fig 3.

A stock solution of 2 M substrate CDNB (MW: 202.55 g/mol) was prepared by dissolving 405.1 mg in 1 ml ethanol. A stock solution of 200 mM GSH cofactor was prepared by dissolving 15.4 mg in 0.25 ml 0.01 M PBS (pH 7.4). After defrosting the rat liver cytosol samples on iced water, they were pooled to obtain pooled rat liver cytosol (PRLC). The GST activity was measured at eight-time intervals: 0, 15, 30, 45, 60, 70, 90, 120 min. Thus, PRLC volumes containing 0.075 mg protein were transferred to eight Eppendorf tubes and 5 μl of the prepared CDNB stock solution (10 mM) was added to each tube. The solutions were completed to 990 μl with 0.01 M PBS (pH 7.4) containing 1 mM EDTA. The prepared solutions were then placed in a shaking water bath adjusted to 37°C for 10 min where the enzymatic reactions started by adding 10 μl GSH stock solution (2 mM). Blanks were prepared using the same compositions of the incubation mixtures except for PRLC, which was replaced by the incubation buffer. The blanks were simultaneously incubated with the incubation mixtures and maintained until the end of the incubation time (120 min). At each time interval, the reaction was ceased by immersing the corresponding Eppendorf tube in ice, and the content of Eppendorf tubes were transferred into 1.5 ml cuvette and the absorbance of the yield product was measured by the UV-visible spectrophotometer at λ_max_ 340 nm. The assay was carried out in duplicate and the average absorbance values were calculated.

### 2.7. Assessment of Bu stability in solvents and biological fluids

Stability of Bu was initially assessed in the experimental solvents including the mobile phase, drug solvent (ACN), and incubation buffer solution 1 mM EDTA in 0.01 M PBS (pH 7.4). After that, Bu metabolic stability was investigated in the biological fluids including rat plasma and liver fractions (S9, microsomes and cytosol). For Bu stability in plasma, incubations were carried out in duplicate and the average Bu concentration was determined. However, in case of Bu stability in liver fractions, 6 assays were performed for each liver fraction and statistical analysis was then carried out accordingly.

#### 2.7.1. Stability of Bu in experimental solvents

The experimental solvents were; buffer solution used in Bu incubation experiments, ACN used for preparing Bu stock solutions, and mobile phase (20 mM NH_4_Ac/MeOH; 20:80, v/v) used for analyzing Bu.

Incubation of Bu in experimental solvents was carried out by preparing 5 μg/ml (20 μM) Bu solutions at final volumes of 1.2 ml. Blanks (containing solvents only) were simultaneously prepared and treated similarly. The procedure was started by incubating the solvents in the shaking water bath at 37°C for 10 min before the addition of Bu or ACN to the blanks. Once the drug was added, aliquots of 100 μl were withdrawn into clean Eppendorf tubes at time intervals of 0, 0.25, 0.5, 1, 2, 3, 4, 6, 23 and 24 h. The samples collected at time intervals of 0 to 6 h, were kept frozen at -80°C and then processed on the next day along with the samples collected at time intervals 23 and 24 h. The samples that were incubated in the mobile phase were spiked with 20 μl IS (10 μg/ml), vortex-mixed for 30 sec, and then 100 μl was transferred to a low volume insert to be analyzed. However, incubated samples in ACN, were spiked with 20 μl IS, vortex-mixed for 30 sec, and then evaporated under a gentle stream of N_2_ gas at 40°C. This was followed by reconstitution with 150 μl mobile phase, vortex-mixed for 30 sec, and then 100 μl was transferred to the low volume insert vial pending analysis using LC-MS/MS.

For processing samples incubated in buffer solution, spiked samples with 20 μl of the IS were vortex-mixed, and extraction of Bu was achieved by adding 1.5 ml *ter*-butyl ethyl ether followed by vortex-mixing for 30 sec. This step was followed by shaking for 15 min using a rotary mixer, adjusted at a medium speed. Then, the tubes were centrifuged at 10000 x *g* for 10 min, and the resulting upper organic layer was separated and evaporated under purified nitrogen gas stream. Reconstitution of samples was carried out by adding 150 μl mobile phase, followed by vortex-mixing and centrifugation at 10,000 x *g* for 10 min. A volume of 100 μl of the supernatant was transferred to the low-volume inserts where 10 μl was injected into the LC-MS/MS system for analysis as described above.

#### 2.7.2. Stability of Bu in biological fluids

Similar to the incubations in the experimental solvents, Bu was incubated in rat plasma samples at final volumes of 1.2 ml. Two Eppendorf tubes were prepared; one contained 5 μg/ml (20 μM) Bu in plasma, while the other contained plasma with ACN to serve as a blank. Plasma samples were processed for analysis applying a previously described LLE method [34].

The assessment of Bu stability in plasma was carried out for a period of 24 h, whereas the same stability was carried out for 2 h in liver fractions. The liver samples were prepared at a final volume of 1.0 ml containing 8 μg/ml (32.5 μM) of Bu. Cofactors solutions were freshly prepared in PBS (pH 7.4) and added to the respective liver fractions. A mixture of cofactors including NADPH, MgCl_2_, UDPGA and GSH were added at a final concentration of 2 mM to samples of pooled rat liver S9 (PRLS9) fractions [46]. Similarly, cofactors including NADPH, MgCl_2_ and UDPGA were added to PRLM fractions, whereas GSH was added to PRLC fractions at the same indicated final concentration described above [47]. Incubations of Bu in microsomes and cytosolic fraction were performed after confirming their enzymatic activities by EROD and GST assays, respectively. The frozen aliquots from the same pooled samples used for EROD and GST assays were thawed and used to assess Bu stability in these fractions. Incubations of Bu in liver fractions were initiated by adding volumes containing 4 mg protein to a 50 μl of the relevant cofactor stock solution (40 mM) in Eppendorf tubes, and then completed to 992 μl with incubation buffer solutions (1 mM EDTA in 0.01 M PBS). The cofactors were similarly added to the respective blank samples of liver fractions. In addition, control samples were also prepared containing the same incubation mixture components except the cofactors, which were replaced by the relevant volume of the buffer solution. The samples were pre-incubated in a shaking water bath at 37°C for 10 min. This was followed by the addition of 8 μl of 1 mg/ml of Bu stock solution at a final concentration of 8 μg/ml. Each cofactor was added at a concentration of 2 mM. Aliquots of 100 μl were withdrawn from the incubated samples at time intervals of 2, 15, 20, 30, 45, 60, 75, 90 and 120 min. The collected samples were processed in spiked plasma samples as described above.

Whether the stability of Bu was evaluated in experimental solvents or biological fluids, calibrators were freshly prepared in the same tested matrix and processed by applying the same corresponding procedure. Consequently, Bu concentrations in the incubated samples were calculated based on the assessed calibrators.

### 2.8. Assessment of Bu stability in solvents and biological fluids in presence of VPA

To investigate the effect of VPA on Bu stability in experimental solvents or biological fluids, VPA was pre-incubated in the matrix before adding Bu in each case. A stock solution of 1 mg/ml of VPA (MW: 144.21 g/mol) was prepared in methanol and kept frozen at -80°C. VPA working solution of 100 μg/ml was prepared by diluting stock solution with ACN. VPA was added to the incubation mixture at the same final molar concentrations of Bu where they were 20 μM (100 μg/ml) in case of experimental solvents and plasma, and 32.5 μM (1 mg/ml) in case of liver fractions. This was achieved by adding 40 μl of VPA working solution (100 μg/ml) to the solvents or plasma incubation mixture, and 5.4 μl of VPA stock solution (1 mg/ml) to liver fractions. Pre-incubation of VPA was carried out for 15 min before adding Bu. The subsequent steps of adding Bu, withdrawing, processing and analyzing the samples were performed as described above for incubating Bu alone.

### 2.9. Statistical analysis

Experimental values were expressed as mean ± SD. Shapiro-Wilk test was performed to assess the normality of the obtained data using GraphPad Prism software (version 9.0.0 (121), San Diego, California, USA). Statistical analysis was performed by using Paired t-test for parametric results, and Wilcoxon matched-pairs signed rank test for nonparametric results. A probability (*P*) value of <0.05 was considered statistically significant.

## 3. Results and discussion

### 3.1. Busulfan analysis conditions

The modified LC-MS/MS method has been applied for Bu quantification using a positive ESI mode (S1 Fig), (34). The tuning parameters had been optimized for the best detection and quantification conditions of both Bu and IS. Separation of the analytes was performed using Symmetry^®^ C_18_ column which resulted in high resolutions of shapes and intensities of the obtained peaks, as well as data reproducibility. The mobile phase improved the sensitivity of the method through enhancement of precursor and product ions formation of both Bu and IS. Furthermore, after applying these experimental conditions, the observed retention time for Bu and IS was approximately 2.01 min (S1–S5 Figs).

### 3.2. Resorufin analysis conditions

The activities of the oxidative enzymes, CYP1A1 in rat liver microsomes were investigated by quantification of resorufin produced from dealkylation process of the substrate 7-ER (Fig 2) by using a modified HPLC-FD method (45), (S6–S8 Figs).

### 3.3. Method validation parameters of both analytical methods

Validation parameters of the modified LC-MS/MS and HPLD-FD methods were evaluated according to the international standards for bio-analytical methods [35, 36].

#### 3.3.1. Linearity and LLOQ

The LC-MS/MS and HPLC-FD analytical methods showed good linearity over the assessed concentration ranges of 1–10 μg/ml for Bu and 10–160 ng/ml for resorufin. The least squares linear regression model revealed good linear correlations (*r* ˃ 0.99) between the response and corresponding analyte concentration with a LLOQ of 1 μg/ml and 10 ng/ml for Bu (S1 Table) and resorufin (S2 Table), respectively.

Moreover, the assessed accuracy and precision at LLOQ was found to be within the acceptable validation limits (RSD% and Bias% ≤20%) for both Bu and resorufin analytical methods. The obtained mean linear regression equation was; *y* = 0.52 + 0.18 *x* (*n* = 6); for Bu analytical method (S1 Table) and *y* = -5.10E^+04^ + 5.15E^+03^ *x* (*n* = 6) for resorufin analytical method (S2 Table), where *y* is the analyte response and *x* is the corresponding analyte concentration in μg/ml for Bu analytical method and ng/ml for resorufin analytical method (S9 and S10 Figs).

#### 3.3.2. Selectivity

The LC-MS/MS and HPLC-FD analytical methods showed capabilities of differentiating between the analyte of interest and other analytes (and/or endogenous molecules). This was demonstrated by the absence of interfering peaks at the retention times of the analytes (Bu or resorufin) or IS after assessing 10 different lots of drug-free rat plasma in case of Bu analytical method and 6 independent lots of rat liver microsomes regarding resorufin analytical method (S3 Fig). This confirms the selectivity of both analytical methods.

#### 3.3.3. Accuracy and precision

The assessed QCs in both methods showed intra/inter-run accuracy and precision within the acceptable limits (bias and RSD% ≤ 15%), (S3 and S4 Tables). In addition, the reported values of these parameters at LLOQ of both methods were complying with the bioanalytical method validation guidelines (≤20%) [35, 36]. The data however, showed adequate accuracy and precision of both methods.

#### 3.3.4. Matrix effect

The potential effect of matrix components on the ionization process of the analyte and the IS was evaluated only for Bu analytical method since resorufin quantification method didn’t involve mass spectrometry [36]. Several approaches had been proposed in order to detect and quantify the matrix effect including post-column infusion and post-extraction analyte spiking [34, 48–50].

#### 3.3.5. Stability

Stability assessment is very crucial parameter for method validation as it ensures that the analyte stability is maintained under various conditions of storage and processing, and hence, the obtained data reflect the actual analyte concentrations directly after sampling [51]. Stability study for LC-MS/MS and HPLC-FD methods was carried out based on the actual applied analytical conditions. Accordingly, stability parameters that were evaluated in analyzing Bu by LC-MS/MS were freeze-thaw, autosampler, and long-term stability. The data on freeze-thaw stability study indicated that Bu was stable for at least five freeze-thaw cycles when stored at -80°C and thawed at room temperature. Autosampler stability (at 25°C) of the processed Bu samples showed that the drug was stable for at least 2 h, covering the actual residential time of the processed samples in the autosampler. The results of long-term stability study of Bu samples (at -80°C) demonstrated that Bu was stable for at least one month without appreciable loss (S5 Table).

On the other hand, based on the actual applied resorufin analytical conditions in which samples were immediately processed and analyzed after the incubation for a total time not exceeding 2 h, short-term stability of the processed samples was evaluated. It was shown that resorufin was stable for at least 2 h under the experimental conditions (S6 Table).

All stability tests were performed using QC samples which were analyzed against calibration curves constructed from freshly spiked standards. After comparing the determined concentrations to the nominal ones, the data have shown to be within the acceptable limits (Bias and RSD%≤15%).

#### 3.3.6. Carryover

The carryover effect is an important validation parameter in bioanalytical method that should be assessed to ensure the accuracy and precision of the obtained data, especially at lower analyte concentrations [52]. In both analytical methods, no carryover effect was observed in all analyzed five lots of blank samples that were injected after ULOQ sample. This finding confirms the reliability of the reported data.

### 3.4. Assessment of the microsomal activity

**Fig 4:**
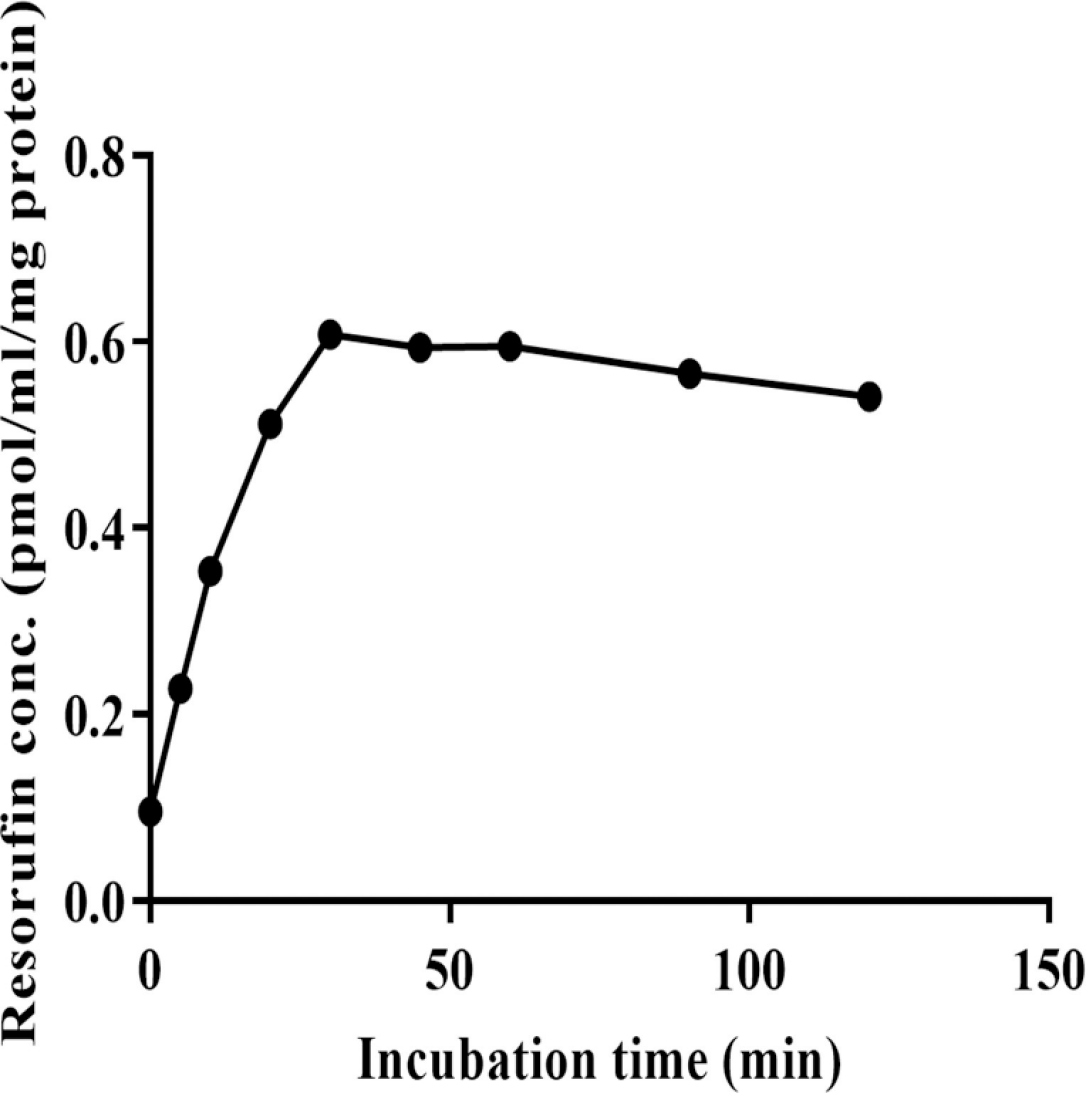
Concentration of resorufin in microsomal fraction versus incubation time. The 7-ER was incubated with 2 mg/ml PRLM containing NADPH, UDPGA and MgCl_2_ at 37°C for 120 min. Each point represents the mean value (n = 2) of two duplicated assays. 7-ER; 7-Ethoxyresorufin, PRLM; Pooled Rat Liver Microsomes, NADPH; Nicotinamide Adenine Dinucleotide Phosphate, UDPGA; Uridine-5`-Diphosphoglucuronic Acid.

For studying the metabolic stability of Bu, the activity of the microsomal CYP1A1 oxidative enzymes was investigated. The PRLM (1 mg/ml) containing CYP1A1 enzyme was incubated with 0.5 μg/ml of 7-ER at 37°C for 120 min. The concentration of the produced resorufin was analyzed using the validated HPLC method. Fig 4 shows a gradual increase in resorufin concentration over a time of 40 min. It has been observed that no change in resorufin level up to 120 min. Therefore, this indicated that CYP1A1 retained its activity for at least 60 min at 37°C.

### 3.5. Assessment of the cytosolic activity

**Fig 5:**
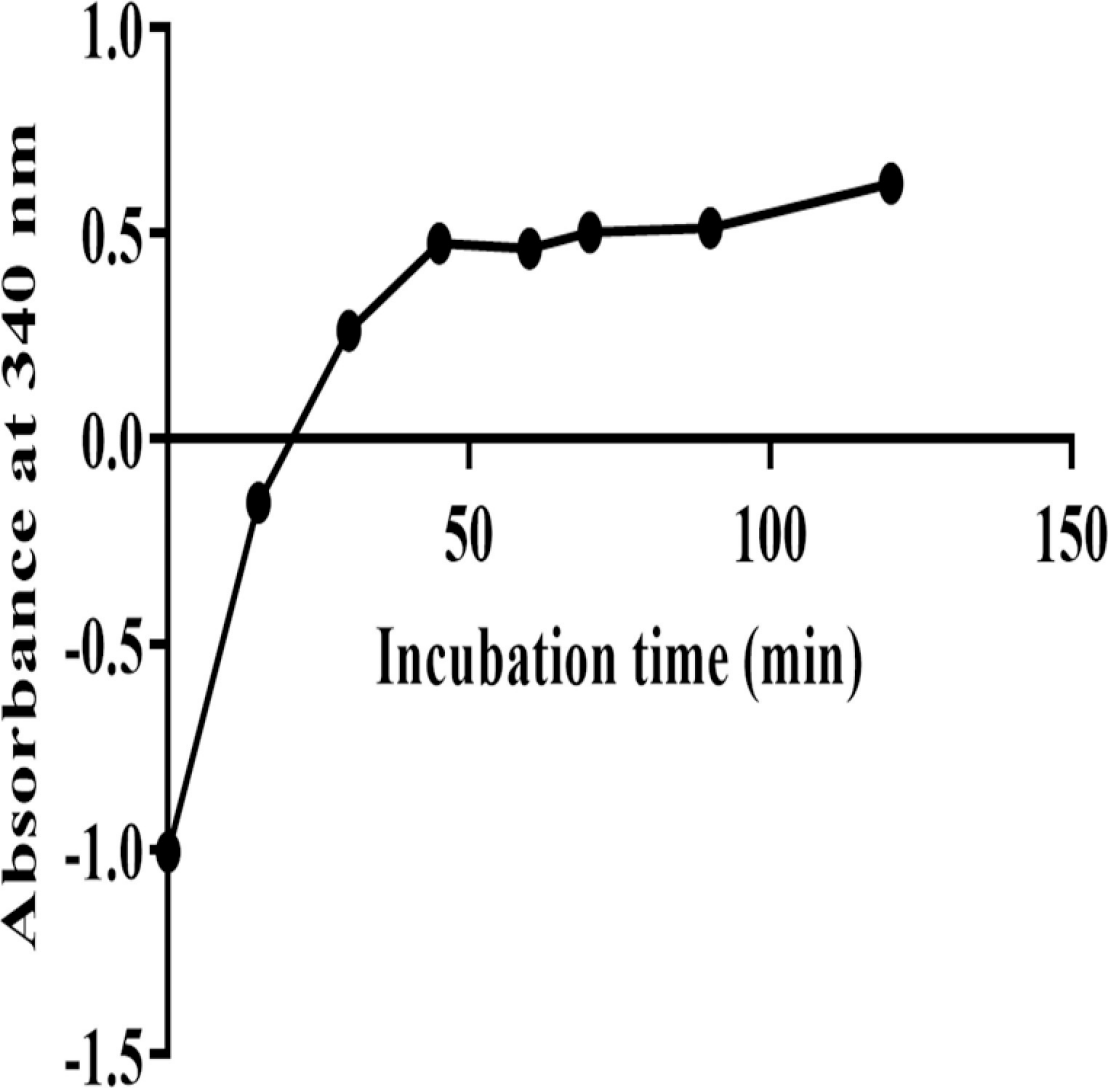
Absorbance of CDNB-GS in cytosolic fraction versus incubation time. The CDNB was incubated with 0.075 mg/ml PRLC containing glutathione at 37°C for 120 min. Each point represents the mean value of two readings from duplicated reactions. CDNB-GS; 1-Chloro-2,4-DinitroBenzen-Glutathione conjugate, PRLC; Pooled Rat Liver Cytosol.

Similarly, for evaluating the metabolic transformation of Bu in cytosol, the metabolic activity of the cytosolic GST enzyme was evaluated. This was carried out by incubating PRLC containing GST with CDNB in presence of GSH at 37°C for 120 min. The carried reaction of GSH conjugation produced dinitrophenyl thioether that was measured at λ_max_ 340 nm. Fig 5 demonstrates an increase in the produced dinitrophenyl thioether over a time of 40 min, after which there was no change in its concentration up to 120 min. This finding confirms that the retained activity of the GST enzyme was for a period of at least 60 min at 37°C.

### 3.6. In vitro assessment of Bu stability in solvents and biological fluids

**Fig 6:**
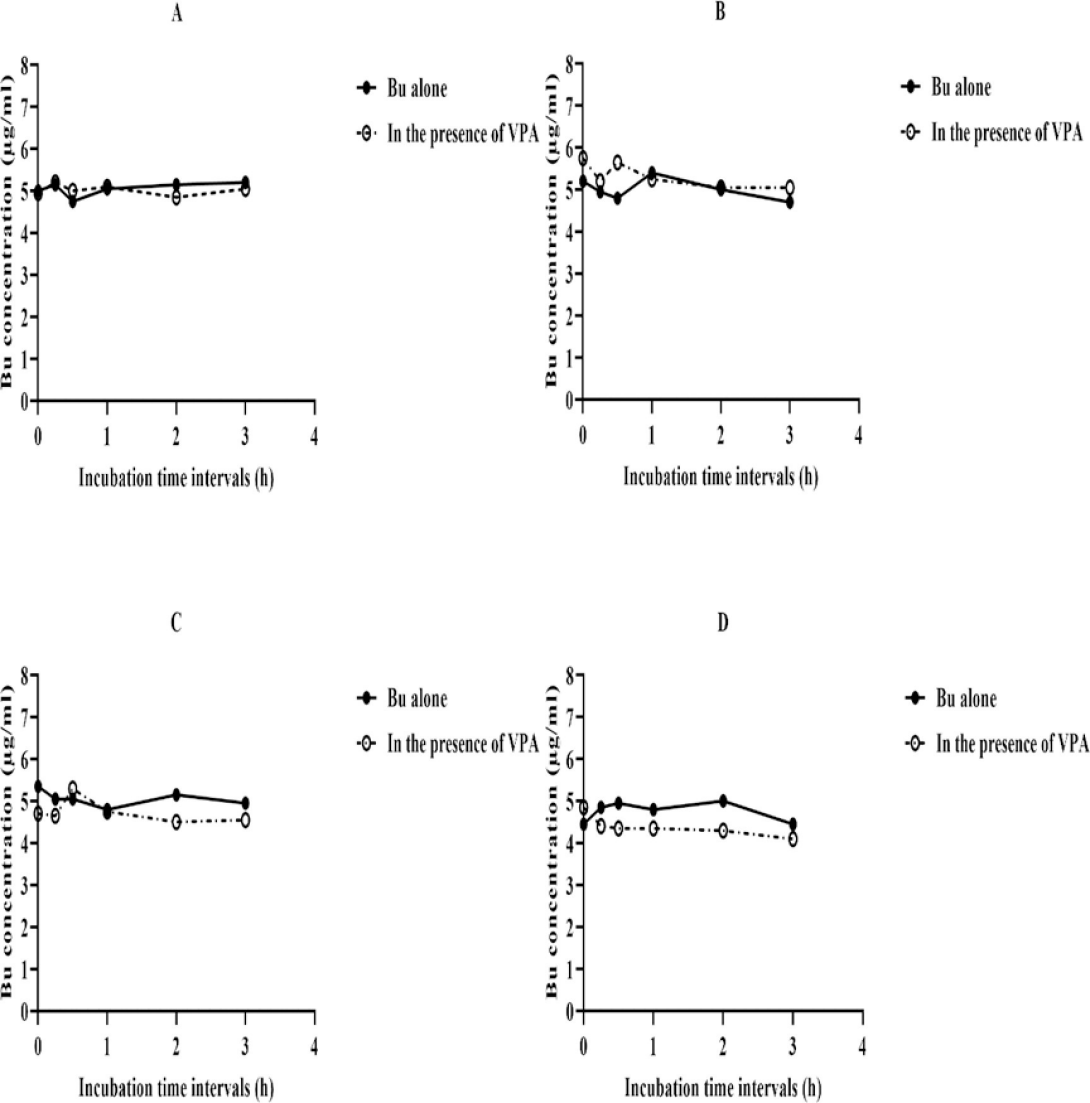
Stability profiles of Bu in the presence and absence of VPA. Bu is incubated alone or in presence of VPA at 37°C for 180 min in (A) mobile phase, (B) ACN, (C) incubation buffer, or (D) rat plasma. Each point represents the average value of duplicated incubations. Bu; Busulfan, VPA; Valproic Acid, ACN; Acetonitrile.

**Fig 7:**
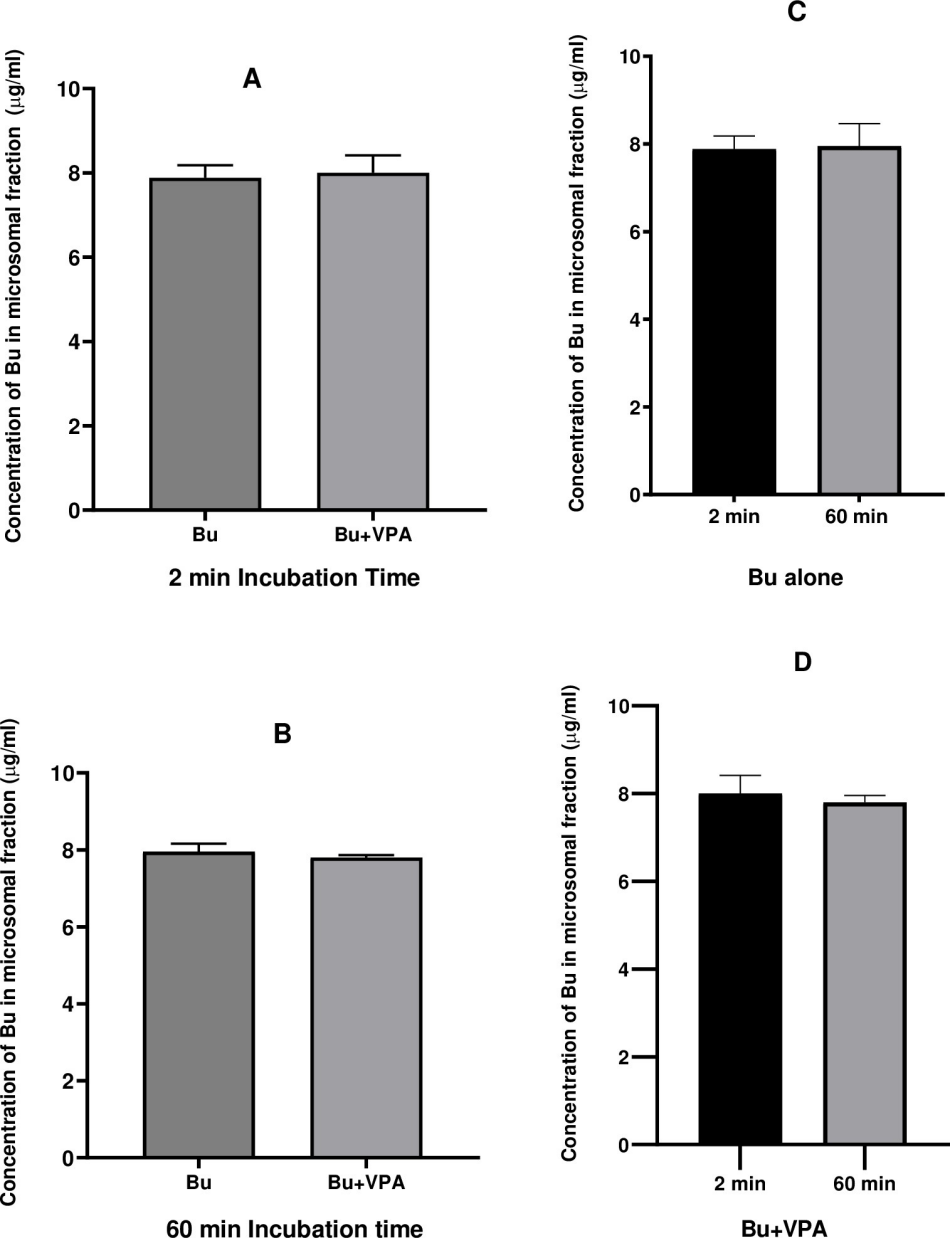
Concentration of Bu incubated in microsomal fraction in presence and absence of VPA. Bu was incubated in PRLM containing NADPH, UDPGA and MgCl_2_ at 37°C for 60 min. The left panel represents analyzed samples for Bu at 2 and 60 min (A and B, respectively) alone and in presence of VPA, while the right panel represents the same analyzed Bu samples alone and in presence of VPA (C and D, respectively) at 2 and 60 min. Each value represents mean ± SD; calculated from 6 assays. Bu; Busulfan, VPA; Valproic Acid, PRLM; Pooled Rat Liver Microsomes, NADPH; Nicotinamide Adenine Dinucleotide Phosphate, UDPGA; Uridine-5`-Diphosphoglucuronic Acid.

**Fig 8:**
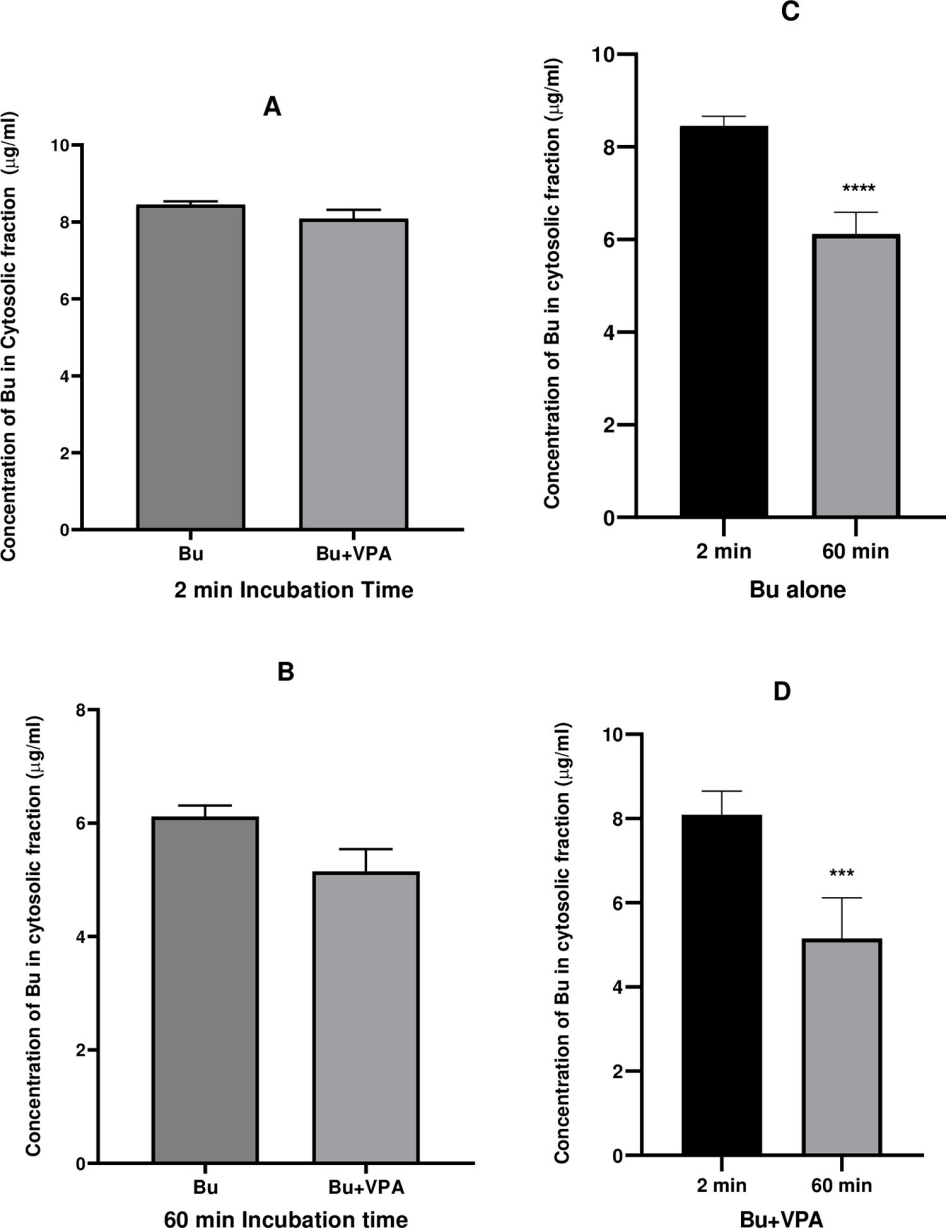
Concentration of Bu incubated in cytosolic fraction in presence and absence of VPA. Bu was incubated in PRLC containing glutathione at 37°C for 60 min. The left panel represents analyzed samples for Bu at 2 and 60 min (A and B, respectively) alone and in presence of VPA, while the right panel represents the same analyzed Bu samples alone and in presence of VPA (C and D, respectively) at 2 and 60 min. Each value represents mean ± SD; calculated from 6 assays. Bu; Busulfan, VPA; Valproic Acid, PRLC; Pooled Rat Liver Cytosol.

**Fig 9:**
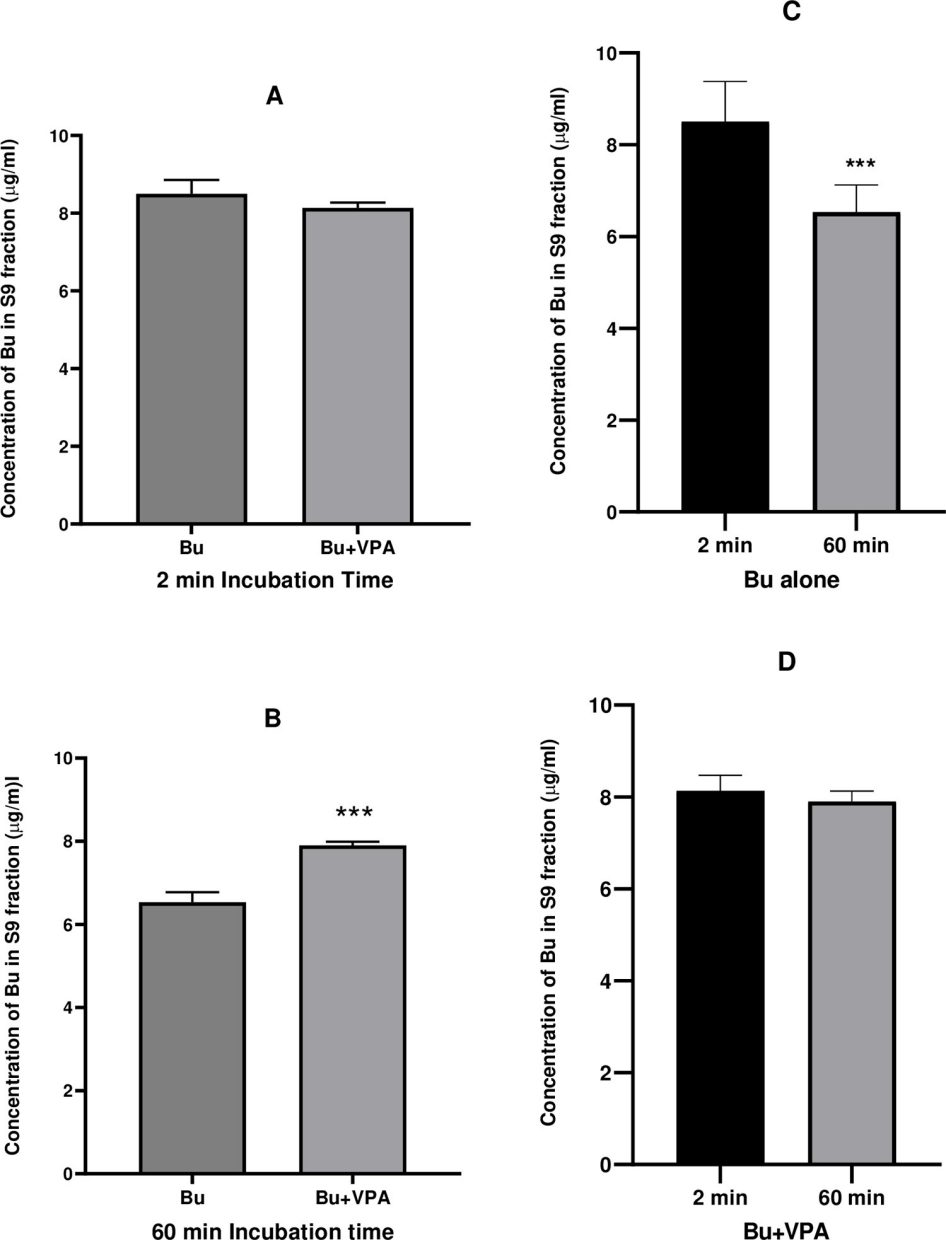
Concentration of Bu incubated in S9 fraction in presence and absence of VPA. Bu was incubated in PRLS9 containing NADPH, UDPGA, MgCl_2_ and glutathione at 37°C for 60 min. The left panel represents analyzed samples for Bu at 2 and 60 min (A and B, respectively) alone and in presence of VPA, while the right panel represents the same analyzed Bu samples alone and in presence of VPA (C and D, respectively) at 2 and 60 min. Each value represents mean ± SD; calculated from 6 assays. Bu; Busulfan, VPA; Valproic Acid, PRLS9; Pooled Rat Liver S9, NADPH; Nicotinamide Adenine Dinucleotide Phosphate, UDPGA; Uridine-5`-Diphosphoglucuronic Acid.

The LC-MS/MS method was applied for the analysis of Bu incubated in the experimental solvents and rat plasma as well as investigating Bu metabolic stability in liver fractions. In each case, investigating of Bu stability was carried out in the absence and presence of VPA (S7 Table). Fig 6 depicts the results of Bu stability when incubated in experimental solvents and plasma. As shown in Fig 6, Bu is stable in these matrices for up to 3 h regardless of VPA presence. Such time duration covers the 2 h incubation period of Bu in liver fractions. Tables 1–3 demonstrate the metabolic stability of Bu alone and in the presence of VPA where incubations were carried out in liver fractions. Similarly, these data were graphically presented in Figs 7–9. As shown in these Figs., there are differences in the metabolic stabilities of Bu within 2 and 60 min in absence and presence of VPA.

**Table 1:** Metabolic stability of Bu alone and in presence of VPA in PRLM.

Incubation time (min)	Bu concentration (μg/ml)			
	Bu alone		In presence of VPA	
	Nominal Conc.	Found Conc. (P-value)	Nominal Conc.	Found Conc. (P-value)
**2**	7.8	7.9 ± 0.3	8.1	8 ± 0.41
**15**	8	7.8 ± 0.46 (0.56)	7.9	8.1 ± 0.39 (0.4)
**20**	7.6	7.8 ± 0.51 (0.76)	8	8.2 ± 0.37 (0.09)
**30**	7.7	7.9 ± 0.34 (0.87)	8	8.1 ± 0.45 (0.22)
**45**	8.2	8 ± 0.29 (0.24)	7.8	8 ± 0.58 (˃0.99)
**60**	8	8 ± 0.52 (0.84)	7.7	7.8 ± 0.15 (0.25)
**75**	7.8	7.7 ± 0.6 (0.3)	7.8	7.7 ± 0.21 (0.2)
**90**	8.1	7.7 ± 0.57 (0.42)	8	8.2 ± 0.33 (0.4)
**120**	7.9	7.6 ± 0.42 (0.18)	8.1	8 ± 0.58 (0.9)

• Incubated Bu concentration was 8 μg/ml in each case.

• Each point represents mean ± SD value of 6 readings obtained from experiments conducted at different occasions.

• Values of control samples are the mean of duplicated readings that were recorded during the first experiment in each case.

• Statistical analysis was performed by applying paired t-test in case of parametric results. However, Wilcoxon matched pairs signed rank test was used for the nonparametric results.

• P-value represents the significance of the difference in found Bu concentration at each time compared to that at the beginning of the incubation period (2 min) in each case.

• P<0.05 is considered statistically significant.

**Table 2:** Metabolic stability of Bu alone and in presence of VPA in PRLC.

Incubation time (min)	Bu concentration (μg/ml)			
	Bu alone		In presence of VPA	
	Nominal Conc.	Found Conc. (P-value)	Nominal Conc.	Found Conc. (P-value)
**2**	8	8.5 ± 0.21	7.7	8.1 ± 0.56
**15**	7.9	7.4 ± 0.28 (0.0022)**	7.8	5.5 ± 0.64 (0.0014)**
**20**	8.2	7.5 ± 0.57 (0.01)*	8	5.4 ± 0.76 (0.0025)**
**30**	7.7	7.4 ± 0.68 (0.0066)**	7.6	5.3 ± 0.56 (0.0012)**
**45**	7.9	6.6 ± 0.84 (0.0035)**	8.2	4.6 ± 0.53 (0.0002)***
**60**	8.1	6.1 ± 0.47 (<0.0001)****	7.9	5.2 ± 0.96 (0.0003)***
**75**	7.8	6.5 ± 0.4 (0.0005)***	7.9	5.2 ± 0.33 (0.0001)***
**90**	8	6.2 ± 0.69 (0.0013)**	7.7	5.2 ± 0.3 (0.03)*
**120**	7.9	6.8 ± 0.24 (0.0002)***	8	5 ± 0.84 (0.0007)***
• Incubated Bu concentration was 8 μg/ml in each case.				
• Each point represents mean ± SD value of 6 readings obtained from experiments conducted at different occasions.				
• Values of control samples are the mean of duplicated readings that were recorded during the first experiment in each case.				
• Statistical analysis was performed by applying paired t-test in case of parametric results. However, Wilcoxon matched pairs signed rank test was used for the nonparametric results.				
• P-value represents the significance of the difference in found Bu concentration at each time compared to that at the beginning of the incubation period (2 min) in each case.				

**P-value**	**Symbol**
˃ 0.05	Non-significant
≤ 0.05	*
≤ 0.01	**
≤ 0.001	***
≤ 0.0001	****

**Table 3:** Metabolic stability of Bu alone and in presence of VPA in PRLS9.

Incubation time (min)	Bu concentration (μg/ml)			
	Bu alone		In presence of VPA	
	Nominal Conc.	Found Conc. (P-value)	Nominal Conc.	Found Conc. (P-value)
**2**	7.7	8.5 ± 0.88	7.8	8.1 ± 0.34
**15**	8.2	7.9 ± 0.65 (0.19)	8.1	8.2 ± 0.42 (0.52)
**20**	8	7.3 ± 0.8 (0.0065)**	7.7	7.6 ± 0.19 (0.024)*
**30**	8.3	7 ± 0.67 (0.0028)**	8.3	8.1 ± 0.73 (0.96)
**45**	7.8	6.8 ± 0.72 (0.0028)**	7.8	8.1 ± 0.45 (0.76)
**60**	7.7	6.5 ± 0.59 (0.0004)***	8	8 ± 0.23 (0.27)
**75**	8	6.7 ± 0.8 (0.0064)**	7.6	8 ± 0.37 (0.35)
**90**	7.9	6.7 ± 0.66 (0.015)*	7.7	7.8 ± 0.26 (0.055)
**120**	8.1	6.4 ± 0.76 (0.0048)**	7.9	7.9 ± 0.78 (0.13)
• Incubated Bu concentration was 8 μg/ml in each case.				
• Each point represents mean ± SD value of 6 readings obtained from experiments conducted on different occasions.				
• Values of control samples are the mean of duplicated readings that were recorded during the first experiment in each case.				
• P-value represents the significance of the difference in found Bu concentration at each time interval compared to that at the beginning of the incubation period (2 min) in each case.				

**P-value**	**Symbol**
˃ 0.05	Non-significant
≤ 0.05	*
≤ 0.01	**
≤ 0.001	***
≤ 0.0001	****

Unlike Bu stability in solvents and rat plasma that were graphically presented from 0 time to 3 h (Fig 6). Figs 7–9 demonstrate Bu stability in liver fractions only for two incubation time intervals; at the beginning of the incubation (2 min) and at 60 min, the time at which Bu is mostly metabolized. The detection at these two-time intervals permits clearly and obviously the determination of any potential inhibition of Bu metabolism as a consequence of VPA pre-incubation. In contrast to a previously reported study, which aimed to assess Bu metabolic profile by showing Bu concentrations at various incubation time intervals [53], the goal of the present work was to investigate the potential DDI between Bu and VPA *in vitro* at two incubation time intervals.

All experimental conditions applied for stability assessment of Bu in solvents and rat plasma samples including the incubation temperature, sampling time intervals and sample processing procedure were kept similar to those employed for incubations in rat liver fractions. The idea was to rule out any factor that might give biased results. Since the obtained chromatographic data indicated stable Bu for at least 2 h in mobile phase, ACN, incubation buffer (pH 7.4) and rat plasma, any subsequent potential Bu degradation in liver fractions could be solely related to the enzymatic activity present in these fractions. The potential Bu enzymatic degradation was confirmed by the simultaneous incubation of control samples that contain components of incubation mixture apart from the cofactors.

In order to minimize variations in Bu metabolism among different rats, liver fractions were pooled from the eight sacrificed rats before incubation on the same day of the experiment. Moreover, since the applied incubation conditions were thought to have appreciable impact on the results of *in vitro* metabolism, attempts were made in order to mimic the *in vivo* conditions with respect to the incubation temperature (37°C) and buffer pH (7.4) [54]. With regard to the concentration of the test drug for *in vitro* experiments, it was recommended to use a little higher than its maximal blood concentration achieved in the *in vivo* study due to stability issue of the drug in the incubation mixture [55]. The maximum Bu blood concentration (C_max_) after being administered intravenously (at a dose of 3.2 mg/kg/day) was found to be around 3.6 μg/ml [56, 57]. Therefore, in this study Bu was incubated at a final concentration of 8 μg/ml in liver fractions, and the calibration curve for Bu determination was ranged from 1 to 10 μg/ml. Furthermore, the concentration of the used solvent (ACN) was kept below 1% in all incubations carried out using liver fractions [58]. The reason behind that is the fact that beyond certain concentrations, it had been demonstrated that several organic solvents including ACN, can extremely disrupt enzymatic activity of *in vitro* models [59, 60].

Although liver S9 sub-cellular fraction is commonly used in drug metabolism assessments, studies demonstrating Bu metabolic stability in this liver fraction are lacking [41, 46, 61–63]. In our present work, we started with S9 fraction since it combines both microsomes and cytosolic fractions providing almost complete presentation of liver metabolic profile with entire phase I and phase II enzymes. Thus, it is a useful confirmatory tool for presence or absence of certain metabolic reactions in intact liver [64]. Furthermore, it was believed that liver S9 is a suitable initial screening model for Bu metabolism where the metabolic fate would be further elucidated in the subdivided two liver fractions. Incubating Bu in rat liver S9 fraction with shaking, for up to 2 h at 37°C, resulted in a gradual decline in Bu concentration with a significant reduction occurred at 60 min (Fig 9A).

Importantly, the incubated control samples which lacked the cofactors did not show such Bu reduction where Bu was stable during the incubation period showing approximately the same initial incubated concentration of 8 μg/ml. Indeed, this finding confirmed that the observed reduction in Bu concentration in the studied samples resulted from the metabolic activity and not due to spontaneous degradation of the drug in the incubation mixture. Furthermore, the application of shaking the incubated samples was shown to have a critical impact on promoting Bu metabolism. This effect has been shown from the absence of Bu metabolism in incubations carried out without shaking. Interestingly, it has been reported that utilization of water bath shaking is a common practice in carrying out *in vitro* incubations in a number of drug metabolic studies [8, 65, 66].

In order to identify whether microsomes or cytosolic fractions were mostly responsible for Bu metabolism and to further ensure the metabolic outcome observed in liver S9 fraction, the same incubation procedure was repeated under the same experimental conditions in microsomes and cytosolic liver fractions. Incubation in microsomes was carried out after conducting EROD assay which confirmed the activity of CYP1A1 subfamily present in this liver fraction, and hence, the viability of the used microsomes. Microsomal EROD assay indicated that the enzymes retained their activity for at least 60 min. (Fig 4). The metabolic stability experiment in rat liver microsomes demonstrated that Bu was stable and didn’t undergo metabolic degradation during the 60 min of the incubation period (Fig 7C). This finding is in agreement with the previously reported findings on Bu metabolic studies involving microsomes [8, 67]. Similarly, the viability of the cytosolic fraction was confirmed by performing GST activity assay prior to studying Bu stability in this liver fraction. This was achieved by assessing the conjugation of GSH to CDNB by the active GST enzyme. The observed increase in spectrophotometric absorbance of the elevated conjugation product (GS-DNB) for 40 min of the incubation time indicated the activity of the GST enzyme in the cytosolic fraction (Fig 5). Thereafter, incubating Bu in rat liver cytosol under the defined incubation conditions caused a significant reduction in the measured Bu concentration from 2 min to 60 min of the incubation time (Fig 8C). This significant reduction in the measured Bu concentration was similarly resulted from incubating the drug in S9 fraction which combines microsomal and cytosolic enzymes (Fig 9C).

Assessing Bu metabolism by GST enzyme through incubation of the drug in liver cytosolic fraction was widely reported in several *in vitro* studies [8, 53, 67–69]. Gibbs and co-workers showed in their innovative study that the intestinal cytosol has approximately similar Bu conjugating activity per milligram of cytosolic protein as that demonstrated by liver cytosol [70]. This actually has largely contributed to the reported inter-individual variability in Bu bioavailability following an oral administration [1]. Moreover, Czerwinski and his team performed a valuable *in vitro* study to characterize the Bu conjugating activity by five GST isoforms, GSTA1-1, GSTA1-2, GSTA2-2, GSTM1-1 and placental GSTP1-1. They concluded that GST is a critical catalyst for Bu conjugation with GSH, and among all of the evaluated isoforms, GSTA1-1 was shown to be the major one responsible for Bu clearance from the body [71].

### 3.7. In vitro assessment of potential Bu and VPA interaction using liver fractions

As indicated above, there are no data available for assessing the potential DDI between Bu and VPA despite their clinical relevance. To the best of our knowledge, the present study is the first evaluation of the potential interaction of VPA and Bu. This was performed by using three well-established *in vitro* liver models. Similarly, the evaluation included the Bu interaction with VPA in experimental solvents and rat plasma, which showed Bu stability for 2 h.

Graphical representations of Bu concentrations when incubated alone and in presence of VPA are presented at 2 min and 60 min in microsomes (Fig 7A and 7B), cytosol (Fig 8A and 8B) and S9 fraction (Fig 9A and 9B). Pre-incubation of VPA in liver microsomes followed by addition of Bu did not show any change in Bu level (Fig 7D) in contrast to its level when incubated alone in the same matrix (Fig 7C). Similarly, pre-incubation of VPA in liver cytosol before Bu addition did not affect the significant reduction in the observed Bu level (Fig 8D) compared to its level when incubated alone in the cytosol (Fig 8C). In contrast, pre-incubating VPA in S9 fraction with VPA before adding Bu results in unchanged Bu level (Fig 9D) compared to the significant reduction in Bu level when incubated alone in the same S9 fraction (Fig 9C). This result indicated a possible inhibition of Bu metabolism by VPA in S9 rat liver fraction, suggesting a potential *in vitro* DDI between Bu and VPA which should further be assessed on different species including humans.

The likely metabolic interaction mechanism between Bu and VPA in the liver S9 fraction could be explained by two possible hypotheses. The first hypothesis is based on the formation of VPA metabolite, *(E)*-2-propyl-2,4-pentadienoic acid or *(E)*-2,4-diene VPA by microsomal CYP450 oxidation reaction. It has been shown that the *(E)*-2,4-diene VPA metabolite has a high affinity for conjugation with GSH by GST action [72, 73]. Moreover, the depletion of GSH by VPA metabolite has been demonstrated both *in vivo* [74, 75] and *in vitro* [76]. In light of the major Bu metabolic pathway, which also involves a conjugation with GSH by the action of the same enzyme, it was suggested that the metabolic inhibition of Bu by VPA may either result from the depletion of GSH stored in the liver or competitive inhibition of GST by *(E)*-2,4-diene VPA, or both. This potential mechanism of metabolic interaction of VPA with Bu requires the presence of the microsomal fraction (containing CYP 450) for the formation of the VPA metabolite, (*(E)*-2,4-diene VPA), and the cytosolic fraction (containing GST) for the conjugation of *(E)*-2,4-diene VPA with GSH. Therefore, the DDI was only observed in liver S9 fraction which combines both fractions, where the cytosolic fraction is only required for Bu metabolism.

The other hypothesis was proposed by El-Serafi and co-workers in a study involving Bu metabolic pathway [12]. Although they did not specifically investigate the DDI between Bu and VPA, they suggested the involvement of microsomal enzymes in Bu metabolic process which seems to be affected by concomitant administration of VPA. The main objective that encouraged them to conduct their study was the fact that many drugs such as phenytoin, itraconazole and metronidazole were reported to interact with Bu and significantly alter its serum concentrations, although they are not substrates of the cytosolic GST, and instead, they are metabolized by microsomal enzymes. El-Serafi and co-workers agreed on the initial step in Bu metabolism which is through conjugation with GSH by GST to form the Bu stable metabolite, THT (Fig 1). Thus, they were interested in their study to investigate the downstream oxidative enzymes responsible for further metabolism of THT including Flavin-containing monooxygenase (FMO_3_) and several CYP450 enzymes. They reported an *in vitro* rapid disappearance of THT when incubated with liver microsomes. The activities of FMO_3_ and CYP450 enzymes were investigated by subsequent incubations of THT with the individual recombinant enzymes. By selectively inhibiting FMO_3_, there was a significant increase in the levels of THT and Bu plasma concentrations in the treated mice. This has been explained by the feedback inhibition of the downstream oxidizing FMO_3_ enzyme by the accumulated THT [12].

Based on these valuable findings, DDI between Bu and VPA in liver S9 fraction could be clearly explained and largely understood. Since several CYP450 isoforms were confirmed to have a role in THT metabolism, inhibition of any of these enzymes could reduce Bu elimination, and thus increase Bu serum levels and the risk of Bu toxicity. In fact, this is the proposed mechanism by which metronidazole, a well-known inhibitor of both CYP2C9 and CYP3A4, was shown to significantly increase Bu serum levels when being used concomitantly [77]. Similarly, VPA was shown to inhibit a number of CYP450 enzymes, including CYP2C9, CYP2C19, and CYP3A4 [26]. All of these enzymes were proved to be involved in the metabolism of the common Bu metabolite, THT [12]. Moreover, the role of CYP2C9, the mostly affected enzyme by VPA inhibition, in overall Bu clearance was shown in an earlier report [11].

Thus, co-administration of VPA may reduce Bu clearance by inhibiting the downstream metabolism of its metabolite THT, possibly through a feedback inhibition. With respect to the current *in vitro* investigation, the proposed mechanism of DDI could not be demonstrated in microsomes or cytosol alone. Since conversion of Bu to THT occurs in cytosol and subsequent THT metabolism and suspected VPA inhibition happen in microsomes, both fractions are required to simultaneously explore such DDI. Therefore, the present study suggests a potential interaction between Bu and VPA in rat liver S9 fraction, which combines both microsomes and cytosolic fraction.

## 4. Conclusions

Bu is a commonly used drug at high dose in the preparative regiments of HSCT. It was shown that a high dose of Bu is associated with generalized seizures, which is usually managed by prophylactic antiepileptic drugs such as VPA. The present study suggested, for the first time, the *in vitro* metabolic interaction of Bu with VPA. Evaluation of Bu levels throughout incubations in three well-established models of subcellular fractions of rat liver was carried out by LC-MS/MS method. The clinically relevant DDI between Bu and VPA was demonstrated by inhibiting Bu metabolism in liver S9 fraction only. Further investigations are warranted to confirm the inhibitory effect of VPA on Bu metabolic transformation in S9 fraction in combination with the metabolic enzyme inhibitors as controls. Due to differential effect in enzyme isoforms and activities, the results of the present study should further be confirmed using human liver fractions. Finally, determination of the mechanism underlying the potential DDI between Bu and VPA, will be followed by investigating the DDI of the two drugs *in vivo*.

## Supporting information

S1 Fig MS (A) and MS-MS (B) scans of Bu as ammonium adduct.
(TIFF)

S2 Fig MS (A) and MS-MS (B) scans of IS (Bu-D8) as ammonium adduct.
(TIFF)

S3 Fig Typical MRM chromatograms of a drug-free rat plasma showing lack of interfering peaks at the retention time of Bu and IS, approximately at 2.01 min. (TIFF)

S4 Fig Typical MRM chromatograms of a drug-free rat plasma supplemented with IS and Bu at LLOQ (1 μg/ml). (TIFF)

S5 Fig Typical MRM chromatograms of a PRLC sample incubated with Bu at a final concentration of 8 μg/ml for 1 h at 37°C. (TIFF)

S6 Fig Typical chromatogram of a drug-free rat liver microsomes showing lack of interfering peaks at resorufin retention time (approximately at 1 min). (TIFF)

S7 Fig Typical chromatogram of a drug-free rat liver microsomes spiked with resorufin at LLOQ (10 ng/ml). (TIFF)

S8 Fig Typical chromatogram of rat liver microsomes incubated with 7-ER (0.5 μg/ml) for 60 min at 37°C. (TIFF)

S9 Fig Representative calibration curve used for quantification of Bu in rat plasma samples. (TIFF)

S10 Fig Representative calibration curve used for resorufin quantification in rat liver microsomes. (TIFF)

S1 Table Linearity data of Bu analytical method. (DOCX)

S2 Table Linearity data of resorufin analytical method. (DOCX)

S3 Table Intra- and inter-run precision and accuracy for quantification of Bu in rat plasma by LC-MS/MS. (DOCX)

S4 Table Intra- and inter-run precision and accuracy of resorufin analytical method by HPLC. (DOCX)

S5 Table Summary of Bu stability in rat plasma samples. (DOCX)

S6 Table Short-term stability of resorufin in rat liver microsomes. (DOCX)

S7 Table Stability of Bu alone and in presence of VPA in (A) mobile phase, (B) ACN, (C) incubation buffer (pH 7.4) and (D) drug-free rat plasma. Values represent the mean of duplicated assays. Incubated Bu concentration was 5 μg/ml.
(DOCX)

## List of abbreviations

can: Acetonitrile
ADEs: Antiepileptic drugs
AUC: Area under plasma concentration-time curve
BSA: Bovine serum albumin
Bu: Busufan
Bu-D8: Deuterated busulfan
CDNB: 1-Chloro-2,4-dinitrobenzene
CML: Chronic myeloid leukemia
Cy: Cyclophosphamide
CYPs: Cytochrome-P450 enzymes
DDIs: Drug-drug interactions
(E)-2,4-diene VPA: (E)-2-propyl-2,4-pentadienoic acid
7-ER: 7-Ethoxyresorufin
EROD: 7-Ethoxyresorufin o-deethylation
ESI: Electrospray ionization
Flu: Fludarabine
FMO: Flavin-containing monooxygenase
GABA: Gamma amino butyrate
GSH: Glutathione
GST: Glutathione-S-transferase
HPLC: High performance liquid chromatography
HPLC-FD: High performance liquid chromatography with fluorescence detector
HSCT: Hematopoietic stem cell transplantation
IV: Intravenous
LC-MS/MS: Liquid chromatography-tandem mass spectrometry
LLE: Liquid-liquid extraction
LLOQ: Lower limit of quantification
MRM: Multiple reaction monitoring
MS: Mass spectrometry
MSD: Male Sprague Dawley®
NADPH: Nicotinamide adenine dinucleotide phosphate
NMDA: N-methyl-D-aspartate
PPT: Protein precipitation
PRLC: Pooled rat liver cytosol
PRLM: Pooled rat liver microsomes
PRLS9: Pooled rat liver S9
PBS: Phosphate buffered saline
QC: Quality control
r: Correlation coefficient
RIC: Reduced-intensity conditioning
RRT: Regimen-related toxicities
RSD: Relative standard deviation
RT: Room temperature
TDM: Therapeutic drug monitoring
THT: Tetrahydrothiophene
THT 1-oxide: Tetrahydrothiophene 1-oxide
UDPGA: uridine 5′-diphosphoglucuronic acid
UGT: Uridine diphosphoglucuronosyl transferase
ULOQ: Upper limit of quantification
UV-Vis spectrophotometer: Ultraviolet-Visible spectrophotometer
VPA: Valproic acid
