# Supplementary figures and images for: Impact of valproic acid on busulfan pharmacokinetics: In vitro assessment of potential drug-drug interaction
Source: PLoS One. 2023 Jan 25;18(1):e0280574. doi: 10.1371/journal.pone.0280574 (PMC9876357; doi:10.1371/journal.pone.0280574)

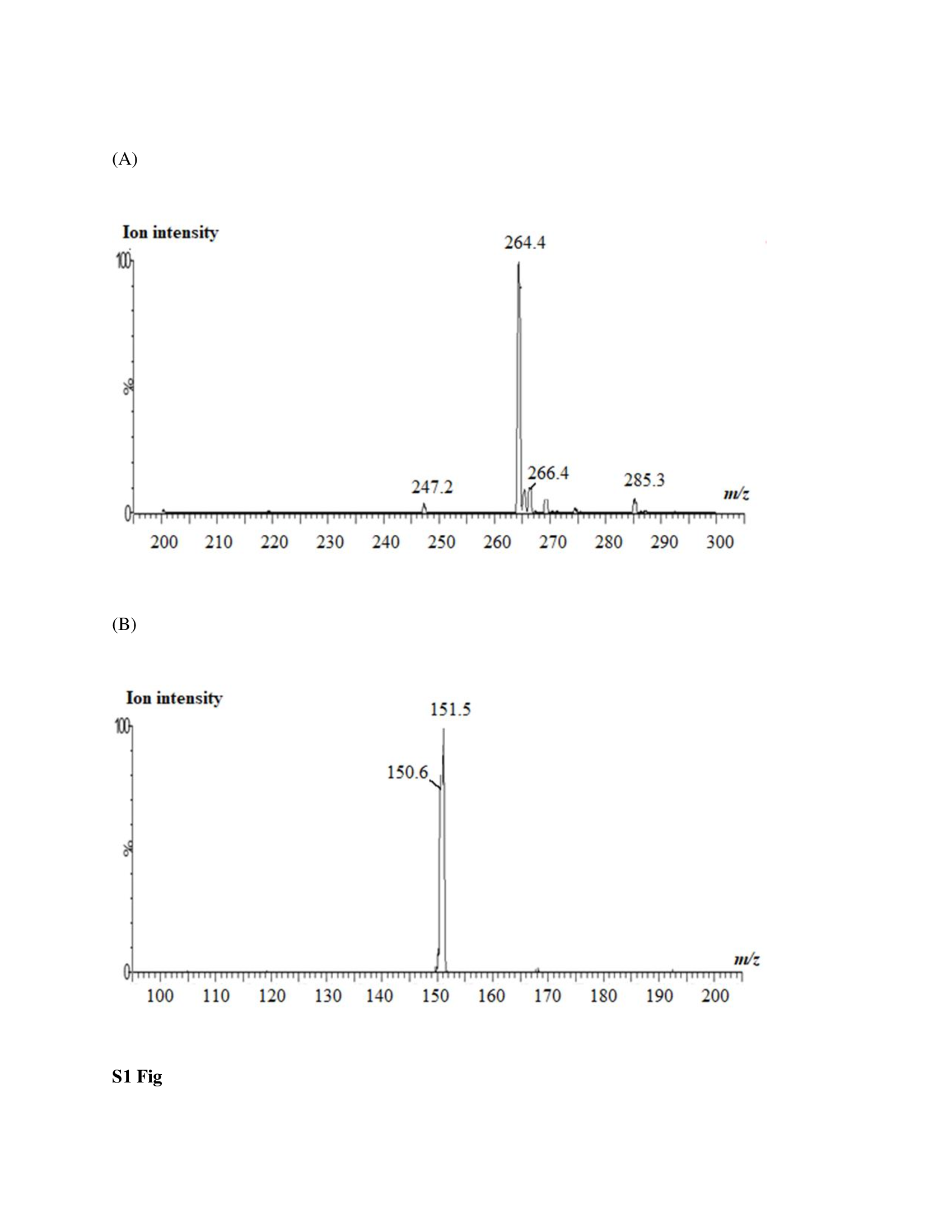

Supplement: S1 Fig — MS (A) and MS-MS (B) scans of Bu as ammonium adduct. (TIFF) [file pone.0280574.s001.tiff]

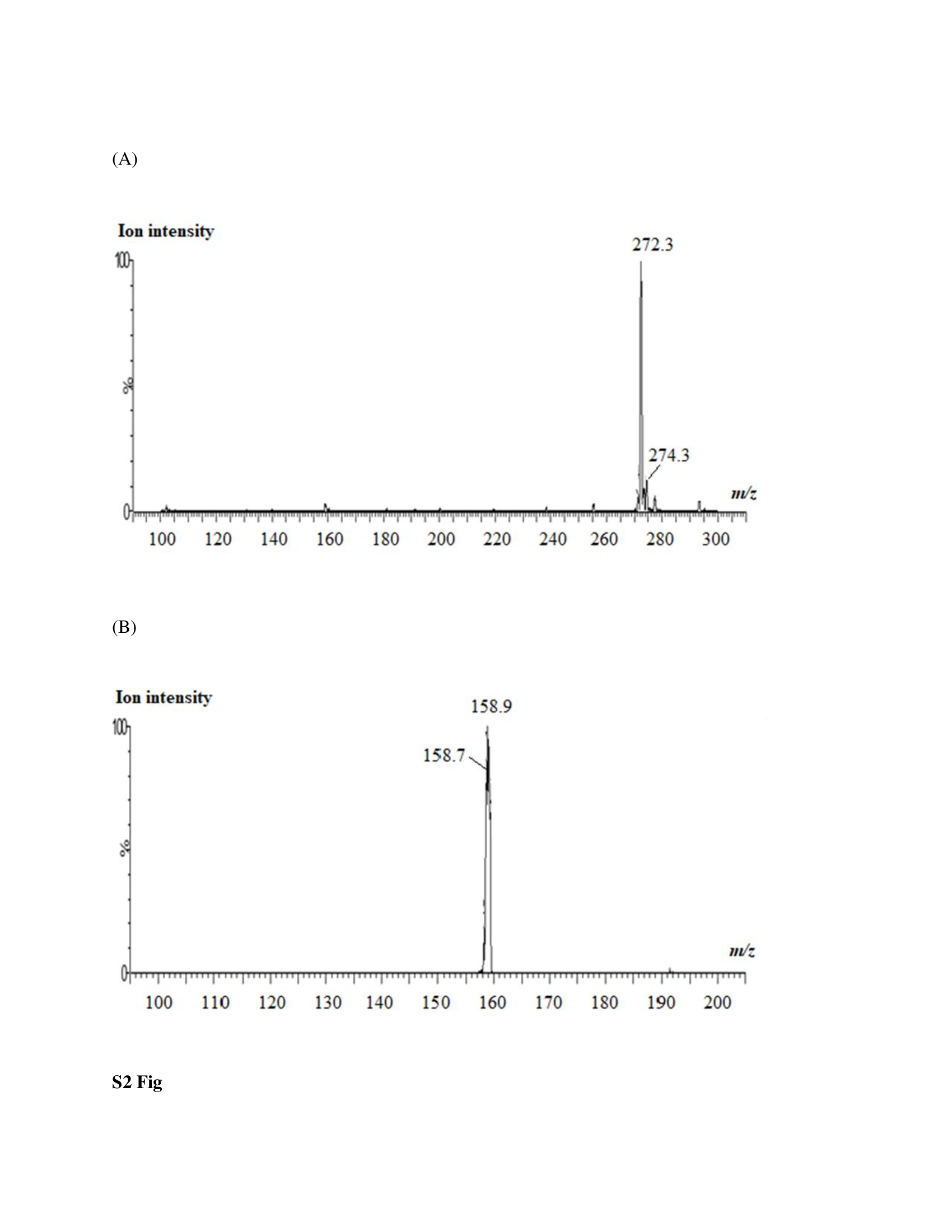

Supplement: S2 Fig — MS (A) and MS-MS (B) scans of IS (Bu-D8) as ammonium adduct. (TIFF) [file pone.0280574.s002.tiff]

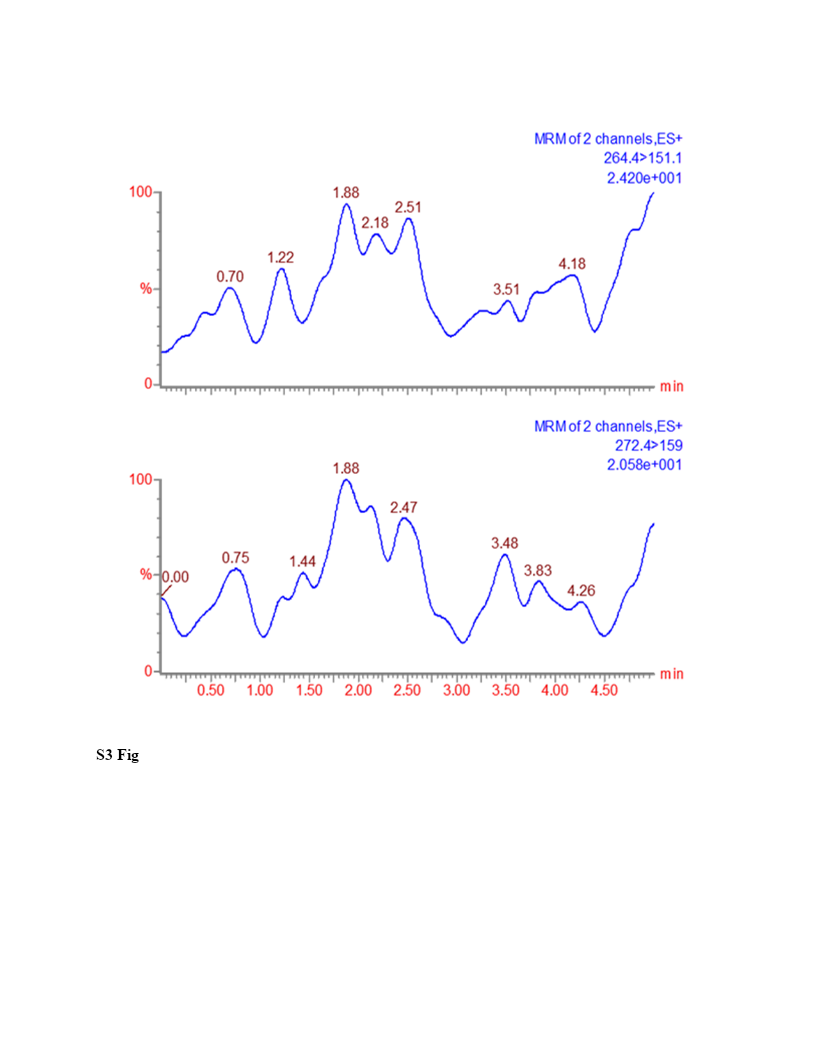

Supplement: S3 Fig — (TIFF) [file pone.0280574.s003.tiff]

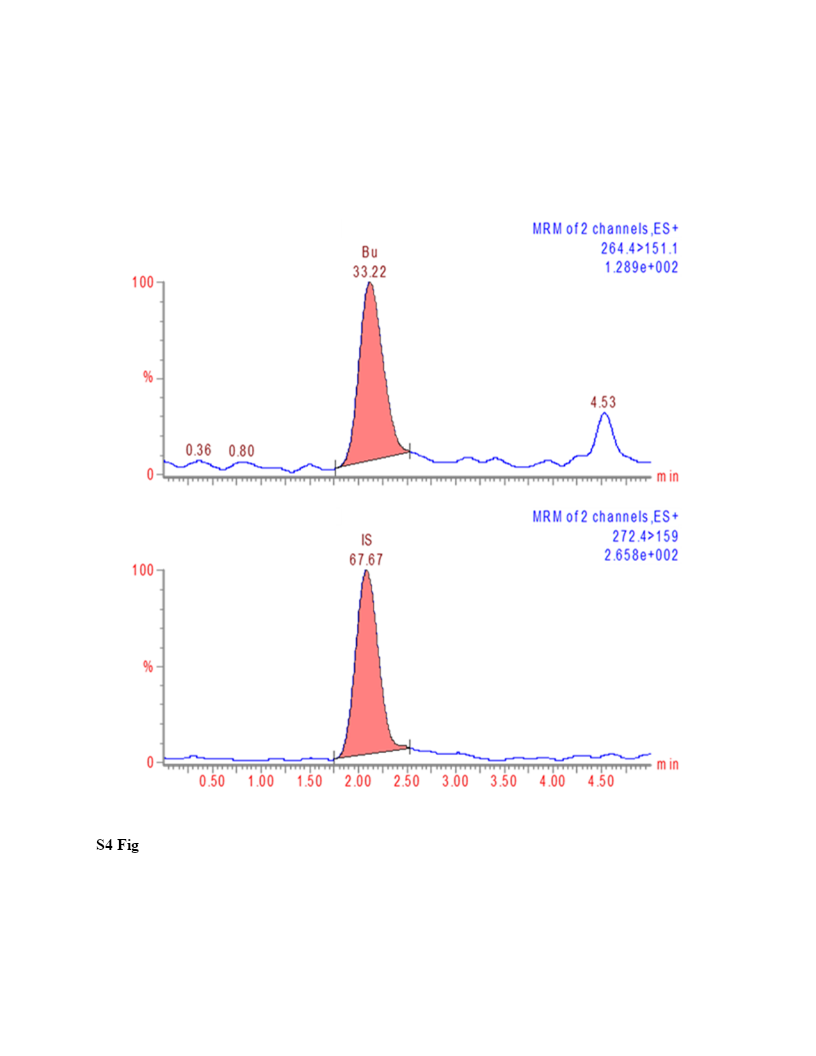

Supplement: S4 Fig — (TIFF) [file pone.0280574.s004.tiff]

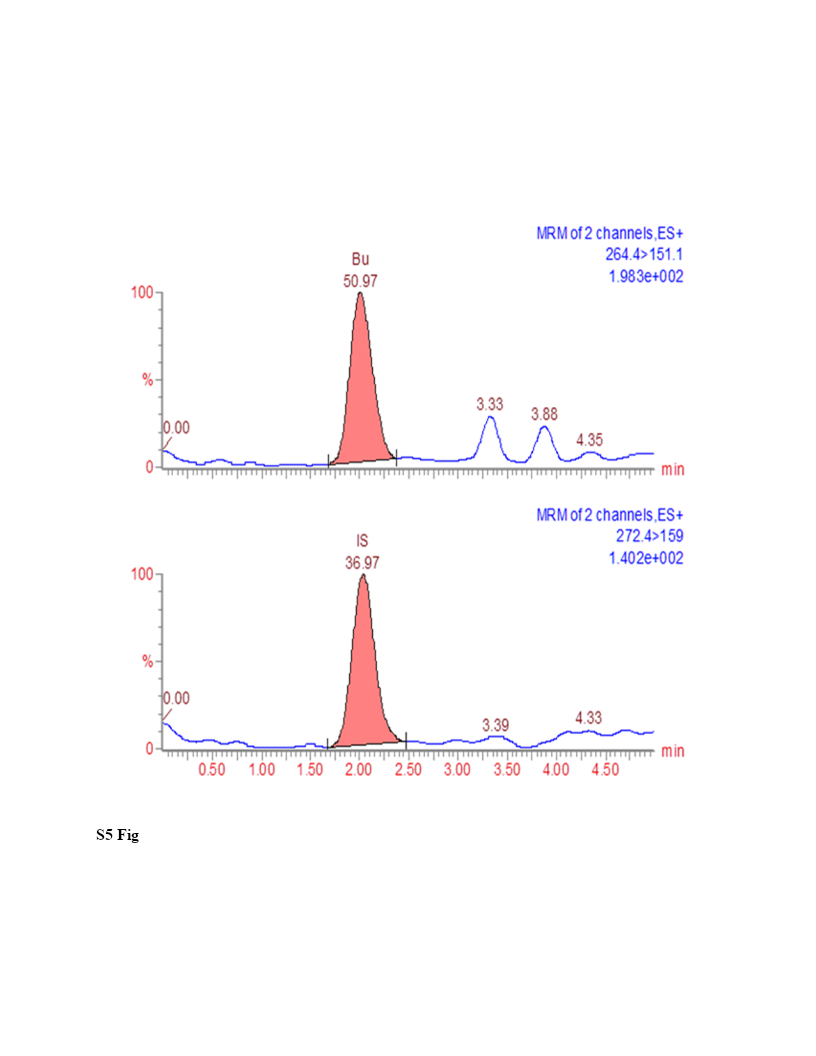

Supplement: S5 Fig — (TIFF) [file pone.0280574.s005.tiff]

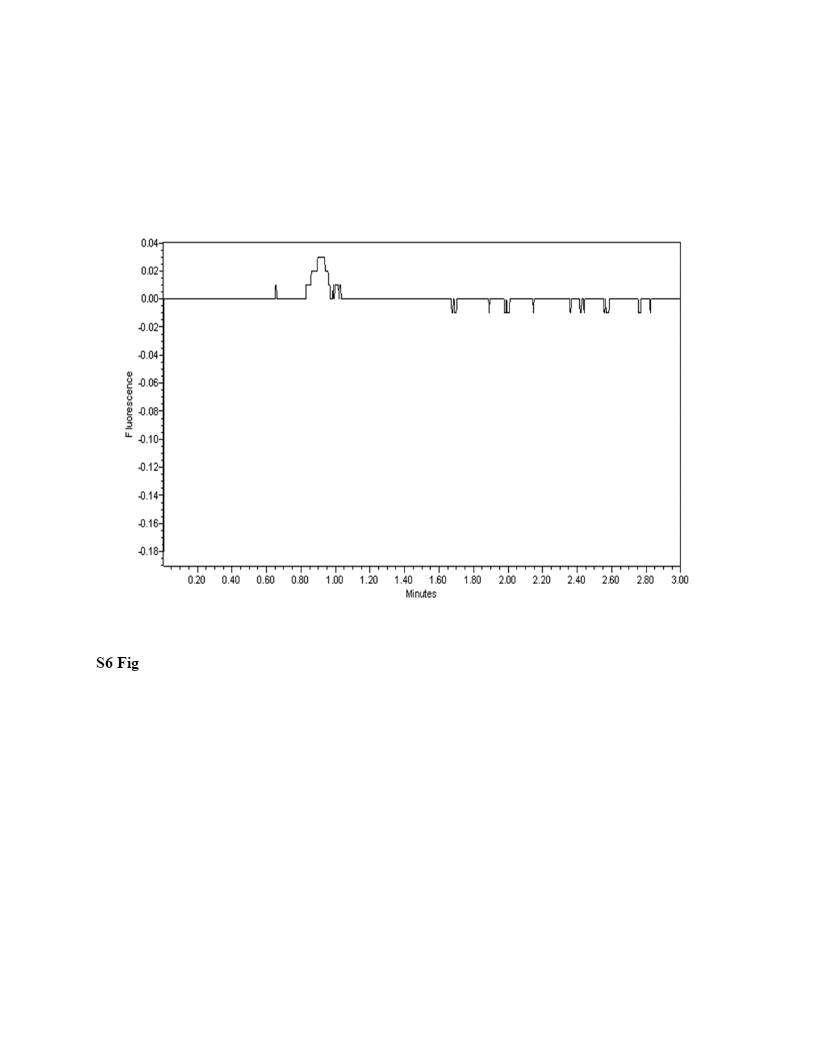

Supplement: S6 Fig — (TIFF) [file pone.0280574.s006.tiff]

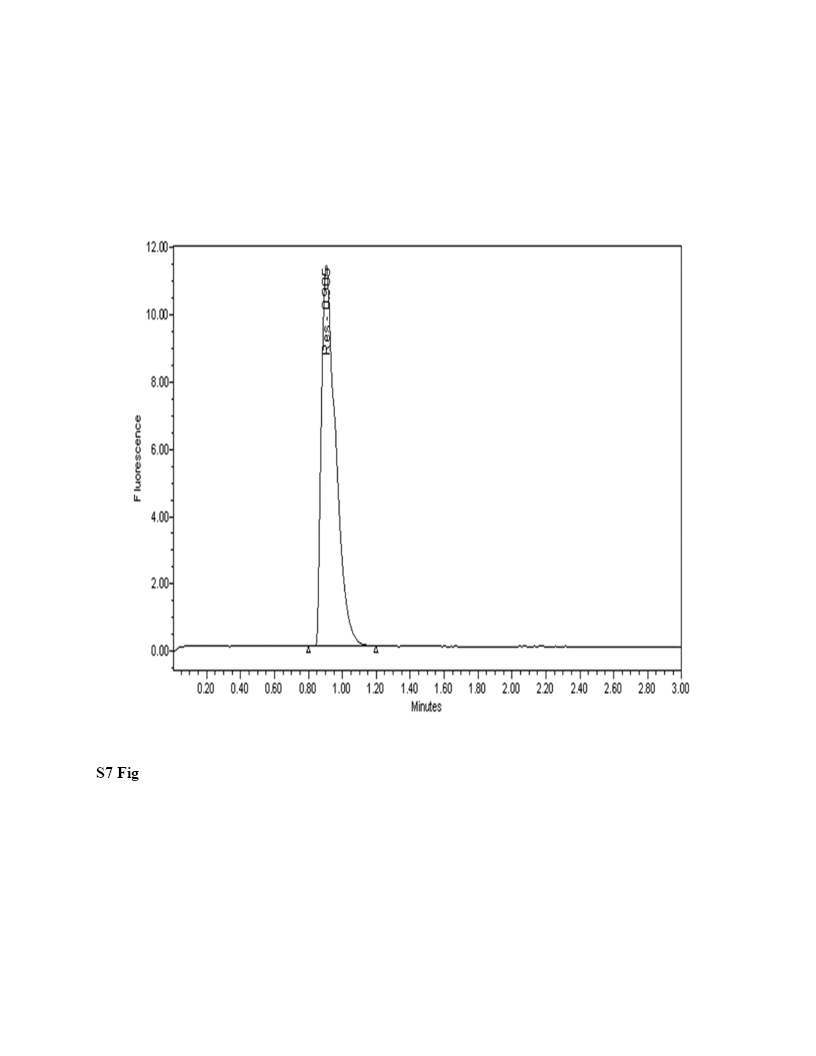

Supplement: S7 Fig — (TIFF) [file pone.0280574.s007.tiff]

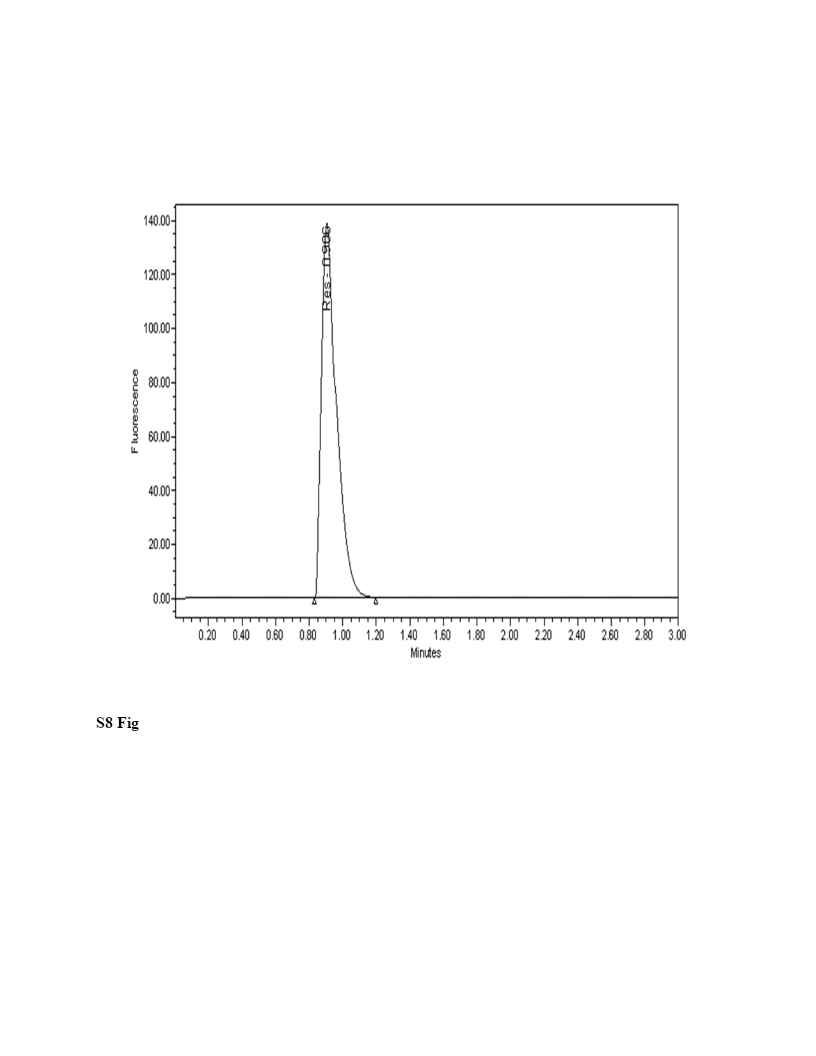

Supplement: S8 Fig — (TIFF) [file pone.0280574.s008.tiff]

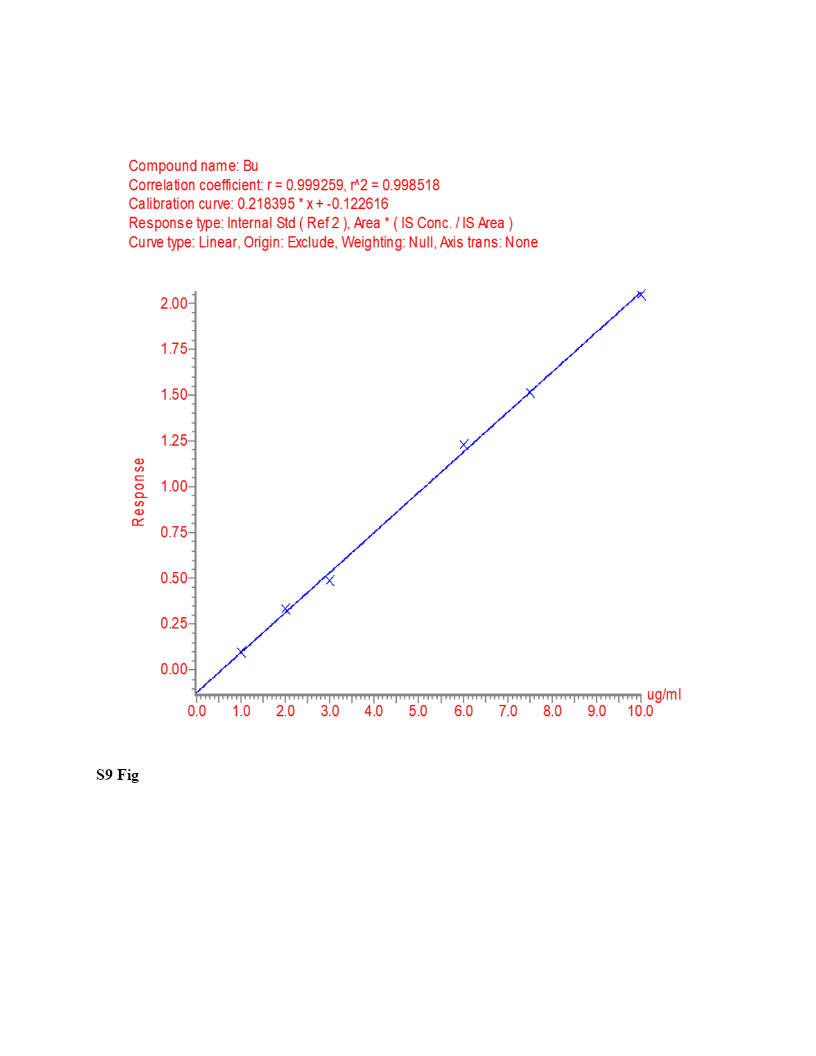

Supplement: S9 Fig — (TIFF) [file pone.0280574.s009.tiff]

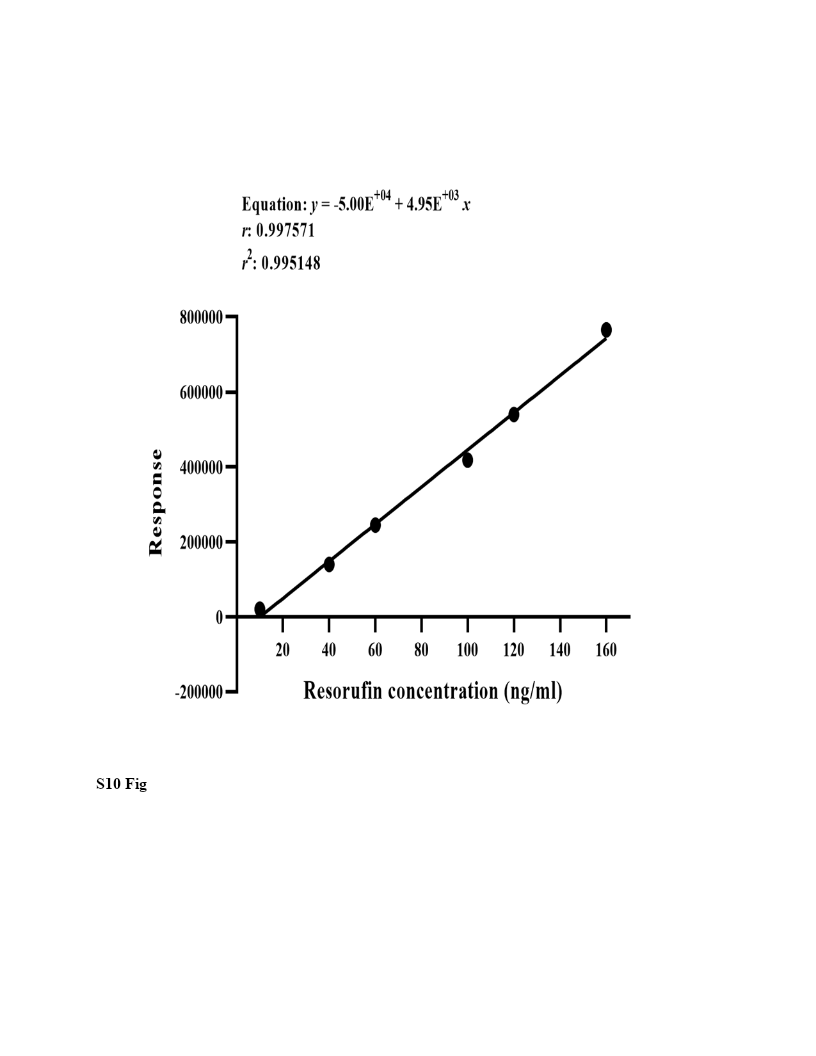

Supplement: S10 Fig — (TIFF) [file pone.0280574.s010.tiff]
