# Supplementary material for: Impact of valproic acid on busulfan pharmacokinetics: In vitro assessment of potential drug-drug interaction
Source: PLoS One. 2023 Jan 25;18(1):e0280574. doi: 10.1371/journal.pone.0280574 (PMC9876357; doi:10.1371/journal.pone.0280574)
Supplement: S1 Table — (DOCX) [file pone.0280574.s011.docx]

**Table 1. Linearity data of Bu analytical method.**

| Serial # | Slope | *y*-Intercept | *r* |
| --- | --- | --- | --- |
| 1 | 0.172605 | 0.560333 | 0.995122 |
| 2 | 0.198012 | 0.62026 | 0.993714 |
| 3 | 0.201553 | 0.635554 | 0.995349 |
| 4 | 0.179856 | 0.403359 | 0.99668 |
| 5 | 0.149265 | 0.526164 | 0.998806 |
| 6 | 0.215817 | 0.385878 | 0.999284 |
| Mean | 0.186185 | 0.521925 | 0.996493 |
| SD | 0.02384 | 0.106434 | 0.002195 |
| RSD% | 12.8 | 20.39 | 0.22 |

- *r* = correlation coefficient
- SD: standard deviation
- RSD: relative standard deviation
- Linear regression equation: *y* = 0.52 + 0.18 *x*; *n*=6, where *y* is the peak area ratio of Bu to IS and *x* is the Bu concentration, expressed as µg/ml
