# Supplementary material for: Impact of valproic acid on busulfan pharmacokinetics: In vitro assessment of potential drug-drug interaction
Source: PLoS One. 2023 Jan 25;18(1):e0280574. doi: 10.1371/journal.pone.0280574 (PMC9876357; doi:10.1371/journal.pone.0280574)
Supplement: S2 Table — (DOCX) [file pone.0280574.s012.docx]

**Table 2. Linearity data of resorufin analytical method.**

| Serial # | Slope | *y*-Intercept | *r* |
| --- | --- | --- | --- |
| 1 | 4.95E^+03^ | -5.65E^+04^ | 0.992752 |
| 2 | 4.91E^+03^ | -4.49E^+04^ | 0.99695 |
| 3 | 4.95E^+03^ | -5.00E^+04^ | 0.997571 |
| 4 | 4.48E^+03^ | -3.47E^+04^ | 0.995136 |
| 5 | 6.55E^+03^ | -6.39E^+04^ | 0.996109 |
| 6 | 5.04E^+03^ | -5.59E^+04^ | 0.992758 |
| Mean | 5.15E^+03^ | -5.10E^+04^ | 0.995212667 |
| SD | 715.34 | 10242.74 | 0.002 |
| RSD% | 13.89 | -20.08 | 0.21 |

- SD: standard deviation
- *r* = correlation coefficient
- Linear regression equation: *y* = -5.10E^+04^ + 5.15E^+03^ *x*; *n* = 6, where *y* is the peak area and *x* is the resorufin concentration, expressed as ng/ml.
