# Supplementary material for: Impact of valproic acid on busulfan pharmacokinetics: In vitro assessment of potential drug-drug interaction
Source: PLoS One. 2023 Jan 25;18(1):e0280574. doi: 10.1371/journal.pone.0280574 (PMC9876357; doi:10.1371/journal.pone.0280574)
Supplement: S3 Table — (DOCX) [file pone.0280574.s013.docx]

**Table 3. Intra- and inter-run precision and accuracy for quantification of Bu in rat plasma by LC-MS/MS.**

| **Nominal concentration (µg/ml)** | **Measured concentration (Mean ± SD) (µg/ml)** | **RSD%** | **Bias%** |
| --- | --- | --- | --- |
| **Intra-run^*^** |  |  |  |
| 1 | 1.14 ± 0.18 | 15.94 | 14 |
| 1.5 | 1.52 ± 0.15 | 9.76 | 1.33 |
| 5 | 4.86 ± 0.3 | 6.27 | -2.8 |
| 8 | 8.44 ± 0.72 | 8.53 | 5.5 |
|  |  |  |  |
| **Inter-run^**^** |  |  |  |
| 1 | 1.04 ± 0.13 | 12.9 | 4 |
| 1.5 | 1.48 ± 0.16 | 11.1 | -1.33 |
| 5 | 5.04 ± 0.32 | 6.37 | 0.8 |
| 8 | 7.92 ± 0.36 | 4.5 | -1 |

- ^*^*n* = 5
- ^**^ Precision and accuracy (bias) were determined from five different runs over a period of one month
- SD: standard deviation
- RSD: relative standard deviation
- RSD (%) = (SD/ Mean) * 100
- Bias (%) = (mean of measured concentration – nominal concentration / nominal concentration) * 100
