# Supplementary material for: Impact of valproic acid on busulfan pharmacokinetics: In vitro assessment of potential drug-drug interaction
Source: PLoS One. 2023 Jan 25;18(1):e0280574. doi: 10.1371/journal.pone.0280574 (PMC9876357; doi:10.1371/journal.pone.0280574)
Supplement: S4 Table — (DOCX) [file pone.0280574.s014.docx]

**Table 4. Intra- and inter-run precision and accuracy of resorufin analytical method by HPLC.**

| **Nominal concentration (ng/ml)** | **Measured concentration (Mean ± SD) (ng/ml)** | **RSD%** | **Bias%** |
| --- | --- | --- | --- |
| **Intra-run^*^** |  |  |  |
| 10 | 11.13 ± 0.11 | 1.02 | 11.3 |
| 20 | 21.76 ± 0.19 | 0.91 | 8.79 |
| 80 | 79.68 ± 0.36 | 0.45 | -0.4 |
| 140 | 139.78 ± 2.9 | 2.08 | -0.16 |
|  |  |  |  |
| **Inter-run^**^** |  |  |  |
| 10 | 10.43 ± 1.43 | 13.72 | 4.3 |
| 20 | 20.54 ± 1.66 | 8.09 | 2.72 |
| 80 | 80.86 ± 6.32 | 7.82 | 1.07 |
| 140 | 140.54 ± 2.32 | 1.65 | 0.38 |

- ^*^n = 5
- ^**^ Precision and accuracy (bias) were determined from five different runs over a period of three weeks.
- SD: standard deviation.
- RSD: relative standard deviation.
- RSD (%) = (SD/ Mean) * 100
- Bias (%) = (mean of measured concentration – nominal concentration / nominal concentration) * 100
