# Supplementary material for: Impact of valproic acid on busulfan pharmacokinetics: In vitro assessment of potential drug-drug interaction
Source: PLoS One. 2023 Jan 25;18(1):e0280574. doi: 10.1371/journal.pone.0280574 (PMC9876357; doi:10.1371/journal.pone.0280574)
Supplement: S5 Table — (DOCX) [file pone.0280574.s015.docx]

**Table 5. Summary of Bu stability in rat plasma samples.**

| **Nominal concentration (µg/ml)** | **1.5** | **5** | **8** |
| --- | --- | --- | --- |
| **Freeze-thaw (*n*=5)** |  |  |  |
| Mean concentration found ± SD | 1.45 ± 0.13 | 4.53 ± 0.36 | 7.8 ± 0.22 |
| RSD% | 8.9 | 7.9 | 2.77 |
| Bias % | -3.33 | -9.5 | -2.5 |
|  |  |  |  |
| **Autosampler at 25°C (2 h) (*n*=5)** |  |  |  |
| Mean concentration found ± SD | 1.6 ± 0.16 | 5.08 ± 0.54 | 7.7 ± 0.6 |
| RSD% | 9.88 | 10.73 | 7.73 |
| Bias% | 6.67 | 1.6 | -3.75 |
|  |  |  |  |
| **Long-term at -80°C (4 weeks) (*n*=6)** |  |  |  |
| Mean concentration found ± SD | 1.57 ± 0.23 | 5.18 ± 0.45 | 7.83 ± 0.77 |
| RSD% | 14.37 | 8.75 | 9.78 |
| Bias% | 4.44 | 3.67 | -2.08 |

- SD: standard deviation.
- RSD: relative standard deviation.
- RSD (%) = (SD/ Mean) * 100
- Bias (%) = (mean of measured concentration – nominal concentration / nominal concentration) * 100
