# Supplementary material for: Impact of valproic acid on busulfan pharmacokinetics: In vitro assessment of potential drug-drug interaction
Source: PLoS One. 2023 Jan 25;18(1):e0280574. doi: 10.1371/journal.pone.0280574 (PMC9876357; doi:10.1371/journal.pone.0280574)
Supplement: S6 Table — (DOCX) [file pone.0280574.s016.docx]

**Table 6. Short-term stability of resorufin in rat liver microsomes.**

| **Nominal concentration (µg/ml)** | **20** | **80** | **140** |
| --- | --- | --- | --- |
| **Autosampler at 25°C (2 h) (*n*=5)** |  |  |  |
| Mean concentration found ± SD | 20.17 ± 1.24 | 79.41 ± 4.89 | 140.58 ± 2.58 |
| RSD% | 6.16 | 6.16 | 1.83 |
| Bias% | 0.84 | -0.73 | 0.42 |

- SD.: standard deviation.
- RSD: relative standard deviation.
- RSD (%) = (SD/ Mean) * 100
- Bias (%) = (mean of measured concentration – nominal concentration / nominal concentration) * 100
