# Supplementary material for: Impact of valproic acid on busulfan pharmacokinetics: In vitro assessment of potential drug-drug interaction
Source: PLoS One. 2023 Jan 25;18(1):e0280574. doi: 10.1371/journal.pone.0280574 (PMC9876357; doi:10.1371/journal.pone.0280574)
Supplement: S7 Table — Stability of Bu alone and in presence of VPA in (A) mobile phase, (B) ACN, (C) incubation buffer (pH 7.4) and (D) drug-free rat plasma. Values represent the mean of duplicated assays. Incubated Bu concentration was 5 μg/ml. (DOCX) [file pone.0280574.s017.docx]

**Table 7. Stability of Bu alone and in presence of VPA in (A) mobile phase, (B) ACN, (C) incubation buffer (pH 7.4) and (D) drug-free rat plasma. Values represent the mean of duplicated assays. Incubated Bu concentration was 5 µg/ml.**

| Tested matrix | Incubation time interval (h) | Found Bu concentration (µg/ml) | |
| --- | --- | --- | --- |
|  |  | **Bu alone** | **In presence of VPA** |
| 1. Mobile phase | 0 | 5 | 4.95 |
|  | 0.25 | 5.15 | 5.2 |
|  | 0.5 | 4.75 | 5 |
|  | 1 | 5.05 | 5.1 |
|  | 2 | 5.15 | 4.85 |
|  | 3 | 5.2 | 5.05 |
|  | 4 | 4.9 | 5.3 |
|  | 6 | 4.85 | 5.1 |
|  | 23 | 5 | 4.7 |
|  | 24 | 5.2 | 4.6 |
| 1. ACN | 0 | 5.2 | 5.75 |
|  | 0.25 | 4.95 | 5.2 |
|  | 0.5 | 4.8 | 5.65 |
|  | 1 | 5.4 | 5.25 |
|  | 2 | 5 | 5.05 |
|  | 3 | 4.7 | 5.05 |
|  | 4 | 5.2 | 5.7 |
|  | 6 | 5.4 | 4.8 |
|  | 23 | 5.05 | 5.45 |
|  | 24 | 5.1 | 5.4 |
| 1. Incubation buffer | 0 | 5.35 | 4.7 |
|  | 0.25 | 5.05 | 4.65 |
|  | 0.5 | 5.05 | 5.3 |
|  | 1 | 4.8 | 4.75 |
|  | 2 | 5.15 | 4.5 |
|  | 3 | 4.95 | 4.55 |
|  | 4 | 4.4 | 4.45 |
|  | 6 | 4.1 | 4.05 |
|  | 23 | 2.1 | 2.1 |
|  | 24 | 2.01 | 1.95 |
| 1. Rat plasma | 0 | 4.45 | 4.85 |
|  | 0.25 | 4.85 | 4.4 |
|  | 0.5 | 4.95 | 4.35 |
|  | 1 | 4.8 | 4.35 |
|  | 2 | 5 | 4.3 |
|  | 3 | 4.45 | 4.1 |
|  | 4 | 4.75 | 3.8 |
|  | 6 | 4.8 | 3.3 |
|  | 23 | 1.75 | 1.6 |
|  | 24 | 1.85 | 1.4 |
